# A complete time-calibrated multi-gene phylogeny of the European butterflies

**DOI:** 10.3897/zookeys.938.50878

**Published:** 2020-06-04

**Authors:** Martin Wiemers, Nicolas Chazot, Christopher W. Wheat, Oliver Schweiger, Niklas Wahlberg

**Affiliations:** 1 Senckenberg Deutsches Entomologisches Institut, Eberswalder Straße 90, 15374, Müncheberg, Germany UFZ – Helmholtz Centre for Environmental Research Halle Germany; 2 UFZ – Helmholtz Centre for Environmental Research, Department of Community Ecology, Theodor-Lieser-Str. 4, 06120, Halle, Germany Senckenberg Deutsches Entomologisches Institut Müncheberg Germany; 3 Department of Biology, Lund University, 22362, Lund, Sweden Lund University Lund Sweden; 4 Department of Biological and Environmental Sciences, University of Gothenburg, Box 461, 405 30, Gothenburg, Sweden University of Gothenburg Gothenburg Sweden; 5 Gothenburg Global Biodiversity Centre, Box 461, 405 30, Gothenburg, Sweden Gothenburg Global Biodiversity Centre Gothenburg Sweden; 6 Department of Zoology, Stockholm University, 10691, Stockholm, Sweden Stockholm University Stockholm Sweden

**Keywords:** Butterflies of Europe, divergence times, macroecology, phylogeny, time tree

## Abstract

With the aim of supporting ecological analyses in butterflies, the third most species-rich superfamily of Lepidoptera, this paper presents the first time-calibrated phylogeny of all 496 extant butterfly species in Europe, including 18 very localised endemics for which no public DNA sequences had been available previously. It is based on a concatenated alignment of the mitochondrial gene COI and up to eleven nuclear gene fragments, using Bayesian inferences of phylogeny. To avoid analytical biases that could result from our region-focussed sampling, our European tree was grafted upon a global genus-level backbone butterfly phylogeny for analyses. In addition to a consensus tree, the posterior distribution of trees and the fully concatenated alignment are provided for future analyses. Altogether a complete phylogenetic framework of European butterflies for use by the ecological and evolutionary communities is presented.

## Introduction

The incorporation of phylogenetic information in ecological theory and research has led to significant advancements by facilitating the connection of large-scale and long-term macro-evolutionary processes with ecological processes in the analysis of species interactions with their abiotic and biotic environments (Webb et al. 2002; Mouquet et al. 2012). Phylogenies are increasingly used across diverse areas of macroecological research (Roquet et al. 2013), such as studies on large-scale diversity patterns (De Palma et al. 2017), disentangling historical and contemporary processes (Mazel et al. 2017), latitudinal diversity gradients (Economo et al. 2018) or improving species area relationships (Mazel et al. 2015). Phylogenetic information has also improved studies on assembly rules of local communities (Cavender-Bares et al. 2009; Gerhold et al. 2015; D’Amen et al. 2018), including spatiotemporal community dynamics (Monnet et al. 2014) and multi-spatial and -temporal context-dependencies (Ovaskainen et al. 2017). Additionally, phylogenetic information has provided insights into the mechanisms and consequences of biological invasions (Knapp et al. 2008; Winter et al. 2009; Li et al. 2015; Gallien et al. 2017). They also contribute to assessments of ecosystem functioning and service provisioning (Díaz et al. 2013; Davies et al. 2016), though phylogenetic relationships cannot simply be taken as a one-to-one proxy for ecosystem functioning (Winter et al. 2013; Mazel et al. 2018). However, they are of great value for studies of species traits and niche characteristics by quantifying the amount of phylogenetic conservatism (Wiens and Graham 2005) and ensuring statistical independence (Kühn et al. 2009) in multi-species studies. Using an ever increasing toolkit of phylogenetic metrics (Schweiger et al. 2008; Tucker et al. 2017), and a growing body of phylogenetic insights, the afore mentioned advances across diverse research fields document how integrating evolutionary and ecological information can enhance assessments of future impacts of global change on biodiversity (Thuiller et al. 2011; Lavergne et al. 2013; Morales-Castilla et al. 2017) and consequently inform conservation efforts (Thuiller et al. 2015; but see also Winter et al. 2013).

Although the amount of molecular data has increased exponentially during the last decades, most available phylogenetic studies are either restricted to a selected subset of species, higher taxa, or to small geographic areas. Complete and dated species-level phylogenetic hypotheses for species-rich taxa of larger regions have been restricted to vascular plants (Durka and Michalski 2012) or vertebrates, such as global birds (Jetz et al. 2012) or European tetrapods (Roquet et al. 2014), or the analyses are based on molecular data from a small subset of species (e.g., 5% in ants; Economo et al. 2018). Regionally complete phylogenetic hypotheses are rare for insects, although they comprise the majority of multicellular life on Earth (Stork 2018), have enormous impacts on ecosystem functioning, provide a multitude of ecosystem services (Noriega et al. 2018), and have long been used as biodiversity indicators (McGeoch 2007).

Here, we present the first comprehensive time-calibrated molecular phylogeny of all 496 extant European butterfly species (Lepidoptera: Papilionoidea), based on one mitochondrial and up to eleven nuclear genes, and the most recent systematic list of European butterflies (Wiemers et al. 2018). European butterflies are well-studied, ranging from population level analyses (Settele et al. 2009) to large-scale impacts of global change (Devictor et al. 2012). There is also good knowledge of species traits and environmental niche characteristics (Bartonova et al. 2014; Schweiger et al. 2014), population trends (van Swaay et al. 2006; van Swaay et al. 2010) and large-scale distributions (Settele et al. 2008; Kudrna et al. 2011). Butterflies are thus well placed for studies in the emerging field of ecophylogenetics (Mouquet et al. 2012).

Compared to other groups of insects, the phylogenetic relationships of butterflies are reasonably well-known, with robust backbone molecular phylogenies at the subfamily (Wahlberg et al. 2005a; Heikkilä et al. 2012; Espeland et al. 2018) and genus-level (Chazot et al. 2019). In addition, molecular phylogenies also exist for most butterfly families (Campbell et al. 2000; Caterino et al. 2001; Wahlberg et al. 2003; Braby et al. 2006; Warren et al. 2008; Wahlberg et al. 2009; Wahlberg et al. 2014; Espeland et al. 2015; Sahoo et al. 2016; Seraphim et al. 2018; Toussaint et al. 2018; Allio et al. 2020) as well as major subgroups (Wahlberg et al. 2005b; Peña et al. 2006; Nylin and Wahlberg 2008; Peña and Wahlberg 2008; Wiemers et al. 2010; Talavera et al. 2013; Peña et al. 2015; Condamine et al. 2018) and comprehensive COI data at the species level are available from DNA barcoding studies (Wiemers and Fiedler 2007; Dincă et al. 2011; Hausmann et al. 2011; Dincă et al. 2015; Huemer and Wiesmair 2017; Litman et al. 2018). Some ecological studies on butterflies have already incorporated phylogenetic information, e.g., on the impact of climate change on abundance trends (Bowler et al. 2015; Bowler et al. 2017), the sensitivity of butterflies to invasive species (Gallien et al. 2017; Schleuning et al. 2016) or the ecological determinants of butterfly vulnerability (Essens et al. 2017). However, the phylogenetic hypotheses used in these studies had incomplete taxon coverage and were not made available for reuse by other researchers. A first complete phylogeny of European butterflies was published by Dapporto et al. (2019) but this tree was not based on a global backbone phylogeny and therefore was also not time-calibrated. To fill these gaps in the literature, and to facilitate the growing field of ecophylogenetics, here we present the first complete and time-calibrated species-level phylogeny of a speciose higher invertebrate taxon above the family level for an entire continent. Importantly, we provide this continent-wide fully resolved phylogeny in standard analysis formats for further advancements in theoretical and applied ecology.

## Materials and methods

### Taxonomic, spatial, and temporal coverage

We analyse a dataset comprising all extant European species of butterflies (Papilionoidea), including the families Papilionidae, Hesperiidae, Pieridae, Lycaenidae, Riodinidae, and Nymphalidae. We base our species concepts, as well as the area defined as Europe, on the latest checklist of European butterflies (Wiemers et al. 2018).

### Acquisition of sequence data

The data were mainly collated from published sources and downloaded from NCBI GenBank (Suppl. material 1). One mitochondrial gene, cytochrome c oxidase subunit I (COI, 1464 bp), was available for all species in the data matrix, in particular the 5’ half of the gene (658 bp, also known as the DNA barcode). Eleven nuclear genes were included when available: elongation factor-1α (EF-1α, 1240 bp), carbamoyl-phosphate synthase domain protein (CAD, 850 bp), cytosolic malate dehydrogenase (MDH, 733 bp), isocitrate dehydrogenase (IDH, 711 bp), glyceraldehyde-3-phosphate dehydrogenase (GAPDH, 691 bp), ribosomal protein S5 (RpS5, 617 bp), arginine kinase (ArgK, 596 bp), wingless (412 bp), ribosomal protein S2 (RpS2, 411 bp), DOPA decarboxylase (DDC, 373 bp), and histone 3 (H3, 329 bp). H3 has been sequenced almost exclusively for the family Lycaenidae, while the other gene regions have been sampled widely also in the other butterfly families. For each gene, the longest available sequence was used. However, in the case of several available sequences of similar length, those of European origin were preferentially used. Sequences were aligned manually to maintain protein reading frame, and were curated and managed using VoSeq (Peña and Malm 2012).

In many cases, new sequences were generated for this study. For these specimens, protocols followed Wahlberg and Wheat (2008) or Wiemers and Fiedler (2007). These include several species that did not have any available published sequences, many of which are island endemics (Table 1). The 239 new sequences have been submitted to GenBank (accessions KC462784–KC462854, MN752702–MN752850, MN829460–MN829496).

Almost all genera are represented by multiple genes, except *Borbo*, *Gegenes*, *Laeosopis*, *Callophrys*, and *Cyclyrius* (the latter recently synonymised with *Leptotes*; Fric et al. 2019) which are represented only by the COI gene. Species represented by only the DNA barcode tend to be closely related to species with more genes sequenced (Suppl. material 1), minimising the potential bias these samples could have in our analyses.

**Table 1.** Newly sequenced species for which no published sequences had previously been available.

Taxon	Origin	COI	EF-1α	GAPDH	Wingless
*Coenonympha orientalis*	Greece	MN829478	MN829462		
*Glaucopsyche paphos*	Cyprus	MN829481	MN829463		
*Gonepteryx maderensis*	Portugal: Madeira	MN829482	MN829464		
*Hipparchia azorina*	Portugal: Azores	MN829483	MN829465		
*Hipparchia bacchus*	Spain: Canary Islands	MN829484	MN829466		
*Hipparchia cretica*	Greece: Crete	MN752718	MN829467	MN752786	MN752837
*Hipparchia gomera*	Spain: Canary Islands	MN829485	MN829468		
*Hipparchia maderensis*	Portugal: Madeira	MN829486			
*Hipparchia mersina*	Greece: Samos	MN752720	MN829469	MN752785	MN752836
*Hipparchia miguelensis*	Portugal: Madeira	MN829487			
*Hipparchia sbordonii*	Italy: Pontine Islands	MN752723			
*Hipparchia tamadabae*	Spain: Canary Islands	MN829488			
*Hipparchia tilosi*	Spain: Canary Islands	MN829489			
*Hipparchia wyssii*	Spain: Canary Islands	MN829490	MN829470		
*Lycaena bleusei*	Spain	MN829492			
*Pieris balcana*	North Macedonia	KC462788			
*Pieris wollastoni*	Portugal: Madeira	KC462820			
*Thymelicus christi*	Spain: Canary Islands	MN829496			

### Phylogenetic tree reconstructions

A biogeographically restricted tree of a given taxon is inherently very asymmetrically sampled. To avoid potentially strong biases when estimating topology and divergence times we chose to build upon the recent genus-level tree of butterflies (Chazot et al. 2019), which provides a well-supported time-calibrated backbone and is congruent with a recent phylogenomic analysis of Lepidoptera (Kawahara et al. 2019). This backbone tree contains 994 taxa, each taxon representing a genus across all Papilionoidea. The tree was time-calibrated using a set of 14 fossil calibration points, which provided minimum ages and ten calibration points based on ages of host plant clades taken from the literature, which provided maximum ages. Importantly, Chazot et al. (2019) tested the robustness of their results to a wide range of alternative assumptions made in the time-calibration analysis, and showed that the estimated times of divergences were robust.

### Analysis overview

To estimate a time-calibrated tree of European butterflies, we first identified the position of the European lineages and designed a grafting procedure accordingly. We split the European butterflies that needed to be added to the tree into 12 subclades. For each of these subclades we combined the DNA sequences of the taxa already included in the backbone to the DNA sequences of the European taxa to assemble an aligned molecular matrix. After identifying the best partitioning scheme, we performed a tree reconstruction without time-calibration (i.e., only estimating branch lengths proportional to relative time). The subclade trees were then rescaled using the ages estimated in the backbone and were subsequently grafted. This procedure was repeated using 1000 trees from BEAST posterior distributions of the backbone and subclade trees in order to obtain a posterior distribution of grafted trees. The details of these procedures are described below.

### Backbone and subclades

The time-calibrated backbone tree provided by Chazot et al. (2019) contained about 55% of all butterfly genera, including 79% of the genera occurring in Europe. A fixed topology was obtained using RAxML (Stamatakis 2014) and node ages where estimated with BEAST v.1.8.3. (Suchard et al. 2018). We used this fixed topology from Chazot et al. (2019) to identify at which nodes European clades should be grafted. We partitioned the analysis into 12 subclades. For each subclade, the DNA sequences of all taxa already included in the global backbone (including also non-European taxa) were combined with the DNA sequences of all the new European taxa that were added. In addition to the focal taxa, we added between two and four outgroups. We note that the relationships of the 12 subclades were fixed according to Chazot et al. (2019), while the relationships of species within the 12 subclades were estimated with the new data.

The subclades, sorted by families, were defined as follows:

Papilionidae – All Papilionidae were placed into one subclade.

Hesperiidae – We identified two main clades to graft within the Hesperiidae: Hesperiinae and Pyrginae. The Hesperiinae subclade was extended to also encompass the subfamilies Heteropterinae and Trapezitinae. The genus *Muschampia*, not available in the backbone, was included in the Pyrginae subclade.

Pieridae – All Pieridae were considered as a single clade.

Lycaenidae – All Lycaenidae were considered as a single clade.

Riodinidae – The only European Riodinidae species, *Hamearis lucina*, was already available in the backbone tree.

Nymphalidae – European Nymphalidae were divided into seven subclades. (i) A subclade for the Apaturinae. (ii) In order to add *Danaus chrysippus* we generated a tree of Danainae. (iii) We combined the sister clades Heliconiinae and Limenitidinae into a single subclade. (iv) Nymphalinae was treated as a single subclade. (v) A first clade of Satyrinae contained the genera *Kirinia*, *Pararge*, *Lasiommata*, *Tatinga*, *Chonala* and *Lopinga*. (vi) A second Satyrinae clade contained the genera *Calisto*, *Euptychia*, *Callerebia*, *Proterebia*, *Gyrocheilus*, *Strabena*, *Ypthima*, *Ypthimomorpha*, *Stygionympha*, *Cassionympha*, *Neocoenyra*, *Pseudonympha*, *Erebia*, *Boerebia*, *Hyponephele*, *Cercyonis*, *Maniola*, *Aphantopus*, *Pyronia*, *Faunula*, *Grumia*, *Paralasa*, *Melanargia*, *Hipparchia*, *Berberia*, *Oeneis*, *Neominois*, *Karanasa*, *Brintesia*, *Arethusana*, *Satyrus*, *Pseudochazara*, and *Chazara*. (vii) A third Satyrinae clade was created for the genus *Coenonympha*. Charaxinae were not treated separately from the backbone. *Charaxes jasius* is the only Charaxinae occurring in Europe and *Charaxes castor* (which is very closely related to *C. jasius*; Aduse-Poku et al. 2009) was already included in the backbone tree from Chazot et al. (2019). Hence, we used the position of *Charaxes castor* for *Charaxes jasius*.

### Partitioning the dataset

For each subclade we ran PartitionFinder 2.1.1 (Lanfear et al. 2016) in order to select the best partitioning strategy and corresponding substitution models. The dataset was initially partitioned into genes and codon positions. Branch lengths were set to linked and the comparison between partitioning strategies was made using the greedy algorithm and BIC score (Lanfear et al. 2012).

### Phylogenetic reconstruction

For each subclade, the dataset was imported in BEAUTi 1.8.3 (Drummond et al. 2012) and partitioned according to the partitioning strategy identified by PartitionFinder. We enforced the monophyly of the clade to be grafted (i.e., excluding the outgroups). All other relationships were estimated by BEAST 1.8.3. (Suchard et al. 2018). We used an uncorrelated relaxed clock with lognormal distribution. By default, we started by setting one molecular clock per partition. If convergence or good mixing could not be obtained after running BEAST we reduced the number of molecular clocks (see details for each dataset further below). We did not add any time-calibration and therefore only estimated the relative timing of divergence. We performed at least two independent runs with BEAST for each subclade. We checked for convergence and mixing of the MCMC using Tracer 1.7.1 (Rambaut et al. 2018) and in the case of full convergence of the runs, the posterior distribution of trees from different runs were combined after removing the burn-in fraction.

### Grafting procedure

Subclades were grafted on the backbone as follows. One backbone was sampled from the posterior distribution of time-calibrated trees from Chazot et al. (2019). For each subclade, one subclade tree was sampled from the posterior distribution of trees, the outgroups removed, and the tree was rescaled based on the crown age of the subclade extracted from the backbone tree. Finally, the rescaled subclade tree was grafted on the backbone after removing all lineages belonging to this subclade in the backbone (i.e., only keeping the stem branch). We repeated this procedure for 1000 backbone trees and 1000 subclade trees, and we thus obtained a posterior distribution of 1000 grafted trees. The topology of the backbone was fixed (see Chazot et al. 2019) but the topologies of the subclades were free. Hence the posterior distribution of grafted trees includes a posterior distribution of topologies and node ages.

We describe below the details of the phylogenetic tree reconstruction for each subclade.

**1. Papilionidae**

*Dataset* – The dataset for the Papilionidae consisted of 36 taxa to which three outgroups were added: *Macrosoma tipulata* (Hedylidae), *Achlyodes busiris* (Hesperiidae), *Pieris rapae* (Pieridae). We concatenated 11 gene fragments (COI, CAD, EF-1α, GAPDH, ArgK, IDH, MDH, RpS2, RpS5, DDC, wingless).

*PartitionFinder* – PartitionFinder identified 12 subsets (Suppl. material 2: Table S1).

*BEAST analysis* – In order to improve the quality of our runs we replaced the default priors for rates of substitutions by uniform prior ranging between 0 and 10 for the following cases: subset5.at, subset5.cg, subset7.cg, subset7.gt, subset12.cg, subset12.gt. We used one molecular clock per subset identified by PartitionFinder and obtained good mixing and convergence. We used a birth-death tree prior. We performed three runs of 40 million generations, sampling trees and parameters every 4000 generations.

*Grafting* – For grafting, the outgroups were removed, as well as *Baronia brevicornis*, the first Papilionidae to diverge and endemic to Mexico (Allio et al. 2020), i.e., we grafted at the most recent common ancestor (MRCA) of all Papilionidae but *Baronia brevicornis*.

**2. Hesperiidae: Hesperiinae**

*Dataset* – The dataset for the Hesperiinae consisted of 169 taxa to which two outgroups were added: *Typhedanus ampyx* (Hesperiidae: Eudaminae), *Mylon pelopidas* (Hesperiidae: Pyrginae). We concatenated 10 gene fragments (COI, CAD, EF-1α, GAPDH, ArgK, IDH, MDH, RpS2, RpS5, wingless).

*PartitionFinder* – PartitionFinder identified 17 subsets (Suppl. material 2: Table S2).

*BEAST analysis* – Preliminary analyses showed problems with the subset 3 (ArgKin_pos3) which was therefore removed from the analyses. In order to improve the quality of our runs we replaced the default priors for rates of substitutions by uniform priors ranging between 0 and 10 for the following case: subset17.cg. The substitution model for the subset 14 was also changed into HKY+I after preliminary analyses. We used one molecular clock per subset identified by PartitionFinder and obtained good mixing and convergence. We used a birth-death tree prior. We performed two runs of 150 million generations, sampling trees and parameters every 15000 generations.

*Grafting* – For grafting, the outgroups were removed and the subclade grafted at the MRCA of Hesperiinae.

**3. Hesperiidae: Pyrginae**

*Dataset* – The dataset for the Pyrginae consisted of 77 taxa to which three outgroups were added: *Typhedanus ampyx* (Hesperiidae: Eudaminae), *Pyrrhopyge zenodorus* (Hesperiidae: Pyrginae), and *Hasora khoda* (Hesperiidae: Coeliadinae). We concatenated ten gene fragments (COI, CAD, EF-1α, GAPDH, ArgK, IDH, MDH, RpS2, RpS5, wingless).

*PartitionFinder* – PartitionFinder identified 14 subsets (Suppl. material 2: Table S3).

*BEAST analysis* – In order to improve the quality of our runs we replaced the default priors for rates of substitutions by uniform priors ranging between 0 and 10 for the following cases: subset7.ac, subset7.gt, subset14.cg, subset3.cg. Preliminary analyses showed problems when using a separate molecular clock for each subset identified by PartitionFinder. We restricted the analysis to one molecular clock. We used a birth-death tree prior. We performed two runs of 100 million generations, sampling trees and parameters every 10000 generations.

*Grafting* – For grafting, the outgroups were removed, and the subclade grafted at the MRCA of Pyrginae.

**4. Pieridae**

*Dataset* – The dataset for the Pieridae consisted of 126 taxa to which three outgroups were added: *Bicyclus anynana* (Nymphalidae), *Achlyodes busiris* (Hesperiidae), and *Papilio glaucus* (Papilionidae). We concatenated eleven gene fragments (COI, CAD, EF-1α, GAPDH, ArgK, IDH, MDH, RpS2, RpS5, DDC, wingless).

*PartitionFinder* – PartitionFinder identified 17 subsets (Suppl. material 2: Table S4).

*BEAST analysis* – In order to improve the quality of our runs we replaced the default priors for rates of substitutions by uniform priors ranging between 0 and 10 for the following case: subset7.cg. The substitution model for the subset 7 was also changed into GTR+G after preliminary analyses. We used one molecular clock per subset identified by PartitionFinder and obtained good mixing and convergence. We used a birth-death tree prior. We performed two runs of 100 million generations, sampling trees and parameters every 10000 generations.

*Grafting* – For grafting, the outgroups were removed, and the subclade grafted at the MRCA of Pieridae.

**5. Lycaenidae**

*Dataset* – The dataset for the Lycaenidae consisted of 187 taxa to which three outgroups were added: *Bicyclus anynana* (Nymphalidae), *Pieris rapae* (Pieridae) and *Hamearis lucina* (Riodinidae). We concatenated 12 gene fragments (COI, CAD, EF-1α, GAPDH, ArgK, IDH, MDH, RpS2, RpS5, DDC, wingless and H3).

*PartitionFinder* – PartitionFinder identified 12 subsets (Suppl. material 2: Table S5).

*BEAST analysis* – In order to improve the quality of our runs we replaced the default priors for rates of substitutions by uniform priors ranging between 0 and 10 for the following cases: subset3.cg, subset6.ag, subset6.at, subset11.gt_subst7.cg. We used one molecular clock per subset identified by PartitionFinder and obtained good mixing and convergence. We used a birth-death tree prior. We performed two runs of 150 million generations, sampling trees and parameters every 15000 generations.

*Grafting* – For grafting, the outgroups were removed, and the subclade grafted at the MRCA of Lycaenidae.

**6. Nymphalidae: Danainae**

*Dataset* – The dataset for the Danainae consisted of 7 taxa to which two outgroups were added: *Euploea camaralzeman* (Nymphalidae: Danainae) and *Lycorea halia* (Nymphalidae: Danainae). We concatenated 9 gene fragments (COI, CAD, EF-1α, GAPDH, IDH, MDH, RpS2, RpS5, wingless).

*PartitionFinder* – PartitionFinder identified eight subsets (Suppl. material 2: Table S6).

*BEAST analysis* – We used one molecular clock per subset identified by PartitionFinder and obtained good mixing and convergence. We used a birth-death tree prior. We performed two runs of 20 million generations, sampling trees and parameters every 2000 generations.

*Grafting* – For grafting, the outgroups were removed, and the subclade grafted at the MRCA of Danainae.

**7. Nymphalidae: Apaturinae**

*Dataset* – The dataset for the Apaturinae consisted of nine taxa to which two outgroups were added: *Timelaea albescens* (Nymphalidae: Apaturinae) and *Biblis hyperia* (Nymphalidae: Biblidinae). We concatenated ten gene fragments (COI, CAD, EF-1α, GAPDH, ArgK, IDH, MDH, RpS2, RpS5, wingless).

*PartitionFinder* – PartitionFinder identified seven subsets (Suppl. material 2: Table S7).

*BEAST analysis* – We used one molecular clock per subset identified by PartitionFinder and obtained good mixing and convergence. We used a birth-death tree prior. We performed two runs of 20 million generations, sampling trees and parameters every 2000 generations.

*Grafting* – For grafting, the outgroups were removed, and the subclade grafted at the MRCA of Danainae.

**8. Nymphalidae: Heliconiinae + Limenitidinae**

*Dataset* – The dataset combined the sister clades Heliconiinae and Limenitidinae and consisted of 92 taxa to which three outgroups were added: *Amnosia decora* (Nymphalidae: Pseudoergolinae), *Apatura iris* (Nymphalidae: Apaturinae) and *Libythea celtis* (Nymphalidae: Libytheinae). We concatenated eleven gene fragments (COI, CAD, EF-1α, GAPDH, ArgK, IDH, MDH, RpS2, RpS5, DDC, wingless).

*PartitionFinder* – PartitionFinder identified 14 subsets (Suppl. material 2: Table S8).

*BEAST analysis* – Preliminary analyses showed problems with the subset 14 (RpS2_pos2) which was therefore removed from the analyses. In order to improve the quality of our runs we replaced the default priors for rates of substitutions by uniform priors ranging between 0 and 10 for the following case: subset7.cg. We used one molecular clock per subset identified by PartitionFinder and obtained good mixing and convergence. We used a birth-death tree prior. We performed two runs of 100 million generations, sampling trees and parameters every 10000 generations.

*Grafting* – For grafting, the outgroups were removed, and the subclade grafted at the split between Limenitidinae and Heliconiinae.

**9. Nymphalidae: Nymphalinae**

*Dataset* – The dataset of Nymphalinae consisted of 83 taxa to which two outgroups were added: *Historis odius* (Nymphalidae: Nymphalinae) and *Pycina zamba* (Nymphalidae: Nymphalinae). We concatenated eleven gene fragments (COI, CAD, EF-1α, GAPDH, ArgK, IDH, MDH, RpS2, RpS5, DDC, wingless).

*PartitionFinder* – PartitionFinder identified 12 subsets (Suppl. material 2: Table S9).

*BEAST analysis* – In order to improve the quality of our runs we replaced the default priors for rates of substitutions by uniform priors ranging between 0 and 10 for the following case: subset5.cg. Preliminary analyses revealed problems when using one molecular clock per subset identified by Partition Finder. We restricted the analysis to one molecular clock for the mitochondrial gene fragments and one molecular clock for the nuclear gene fragments. We used a birth-death tree prior. We performed two runs of 100 million generations, sampling trees and parameters every 10000 generations.

*Grafting* – For grafting, the outgroups were removed, and the subclade grafted at the MRCA of Nymphalinae.

**10. Nymphalidae: Satyrinae 1**

*Dataset* – The first Satyrinae dataset consisted of 13 taxa, belonging to the genera *Kirinia*, *Pararge*, *Lasiommata*, *Tatinga*, *Chonala*, and *Lopinga*, to which three outgroups were added: *Bicyclus anynana* (Nymphalidae: Satyrinae), *Acrophtalmia leuce* (Nymphalidae: Satyrinae), and *Ragadia makuta* (Nymphalidae: Satyrinae). We concatenated 5 gene fragments (COI, EF-1α, GAPDH, RpS5, wingless).

*PartitionFinder* – PartitionFinder identified six subsets (Suppl. material 2: Table S10).

*BEAST analysis* – We used one molecular clock per subset identified by PartitionFinder and obtained good mixing and convergence. We used a birth-death tree prior. We performed two runs of 20 million generations, sampling trees and parameters every 2000 generations.

*Grafting* – For grafting, the outgroups were removed, and the subclade grafted at the crown of the clade after removing the outgroups.

**11. Nymphalidae: Satyrinae 2**

*Dataset* – The second Satyrinae dataset consisted of 161 taxa, belonging to the genera *Calisto*, *Euptychia*, *Callerebia*, *Proterebia*, *Gyrocheilus*, *Strabena*, *Ypthima*, *Ypthimomorpha*, *Stygionympha*, *Cassionympha*, *Neocoenyra*, *Pseudonympha*, *Erebia*, *Boerebia*, *Hyponephele*, *Cercyonis*, *Maniola*, *Aphantopus*, *Pyronia*, *Faunula*, *Grumia*, *Paralasa*, *Melanargia*, *Hipparchia*, *Berberia*, *Oeneis*, *Neominois*, *Karanasa*, *Brintesia*, *Arethusana*, *Satyrus*, *Pseudochazara*, and *Chazara*, to which three outgroups were added: *Coenonympha pamphilus* (Nymphalidae: Satyrinae), *Taygetis virgilia* (Nymphalidae: Satyrinae), and *Pronophila thelebe* (Nymphalidae: Satyrinae). We concatenated ten gene fragments (COI, CAD, EF-1α, GAPDH, ArgK, IDH, MDH, RpS2, RpS5, wingless).

*PartitionFinder* – PartitionFinder identified eleven subsets (Suppl. material 2: Table S11).

*BEAST analysis* – In order to improve the quality of our runs we replaced the default priors for rates of substitutions by uniform prior ranging between 0 and 10 for the following cases: subset5.ac, subset5.ag, subset5.at, subset5.cg, subset5.gt. We used one molecular clock per subset identified by PartitionFinder and obtained good mixing and convergence. We used a birth-death tree prior. We performed two runs of 100 million generations, sampling trees and parameters every 10000 generations.

*Grafting* – For grafting, the outgroups were removed, and the subclade grafted at the crown of the clade after removing the outgroups.

**12. Nymphalidae: Satyrinae 3**

*Dataset* – The third Satyrinae dataset consisted of 15 taxa all belonging to the genus *Coenonympha*, to which two outgroups were added: *Sinonympha amoena* (Nymphalidae: Satyrinae) and *Oressinoma sorata* (Nymphalidae: Satyrinae). We concatenated nine gene fragments (COI, CAD, EF-1α, GAPDH, IDH, MDH, RpS2, RpS5, wingless).

*PartitionFinder* – PartitionFinder identified six subsets (Suppl. material 2: Table S12).

*BEAST analysis* – We used one molecular clock per subset identified by PartitionFinder and obtained good mixing and convergence. We used a birth-death tree prior. We performed two runs of 20 million generations, sampling trees and parameters every 2000 generations.

*Grafting* – For grafting, the outgroups were removed, and the subclade grafted at the crown of *Coenonympha*.

### Quality control

Species identities of the chosen sequences for the dataset were validated by blasting the DNA barcode sequences against the Barcode Of Life Database (http://www.boldsystems.org/), which has a good representation of European butterfly species due to a number of barcoding projects implemented in different countries (e.g., Wiemers and Fiedler 2007; Dincă et al. 2011; Hausmann et al. 2011; Dincă et al. 2015; Huemer and Wiesmair 2017; Litman et al. 2018). In almost all cases, the sequences came from the same voucher specimen itself. In 17% of cases (Suppl. material 1), the sequences used were from different individuals. In these cases special care was taken to use sequences from reliable sources, preferably those with voucher photographs.

We estimated our time-calibration from a recent re-evaluation of the timing of divergence of higher-level Papilionoidea. We used the topology inferred by Chazot et al. (2019) as a backbone in our grafting procedure. This topology was fixed in Chazot et al. (2019), hence only node ages were estimated. However, within each subclade we grafted, we let BEAST estimate the topology in addition to node ages. Several sections of the European butterfly tree remain poorly supported. This most likely arises from the lack of molecular information as well as recent and rapid diversification events within *Polyommatus*, *Hipparchia*, or *Pseudochazara* for example. Further more detailed work is needed in these groups, building on preliminary studies (e.g., Wiemers and Fiedler 2007; Vila et al. 2010; Wiemers et al. 2010; Verovnik and Wiemers 2016; Vishnevskaya et al. 2016), which might show that some of the taxa need to be synonymised (as e.g., *Erebia polaris* with *E. medusa*; see Aarvik et al. 2017). Most of the higher relationships among genera are well supported, however. Exceptions with low support values are the relationships among the genera *Anthocharis*, *Euchloe*, and *Zegris* (Pieridae: Pierinae), among *Agriades*, *Cyaniris*, *Eumedonia*, *Kretania* and *Plebejidea* (Lycaenidae: Polyommatinae), some relationships among the Theclinae (Lycaenidae) and between *Aphantopus* and *Pyronia* (Nymphalidae: Satyrinae). This also means that the apparent non-monophyly of the genera *Euchloe*, *Kretania*, *Satyrium*, and *Pyronia* in our tree needs to be confirmed by further studies. The only subfamily relationship with low support is the sister relationship of Aphnaeinae with Lycaeninae. In Dapporto et al. (2019)Aphnaeinae turned out as sister to the remaining Lycaenidae, a result in line with Espeland et al. (2018), although with low support in the latter study. In most of these cases, the low support values are due to insufficient molecular information for those groups of taxa.

We show here a synthetic tree summarising the posterior distribution of topologies and node ages, but the posterior distribution of grafted trees can be found in the Supporting Information, providing a distribution of alternative topologies and node ages estimated by BEAST. We strongly advise any researcher using these phylogenetic trees to repeat any analyses on at least 100 trees randomly sampled from this posterior distribution in order to account for topology and node age uncertainties. This tree can also help to identify the sections of the tree lacking molecular information and therefore points at the sections that should be targeted in the future when generating new molecular data.

### Dataset descriptions

The analysed dataset (a concatenated alignment of the genes COI, CAD, EF-1α, GAPDH, ArgK, IDH, MDH, RpS2, RpS5, DDC, wingless, and H3) is available in NEXUS format and the posterior distribution of ML trees and the consensus tree in NEWICK format at DOI: https://dx.doi.org/10.5281/zenodo.3531555.

## Conclusions

**Figure 1.**
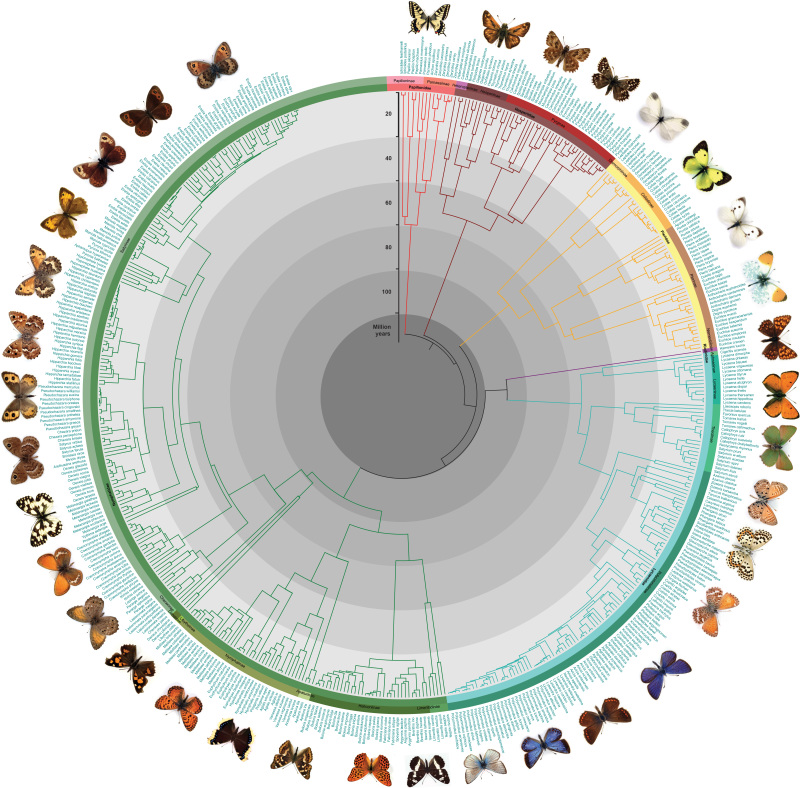
Time-calibrated tree of European butterflies (Lepidoptera: Papilionoidea) with time scale and taxonomic assignment to subfamilies and families.

**Figure 2.**
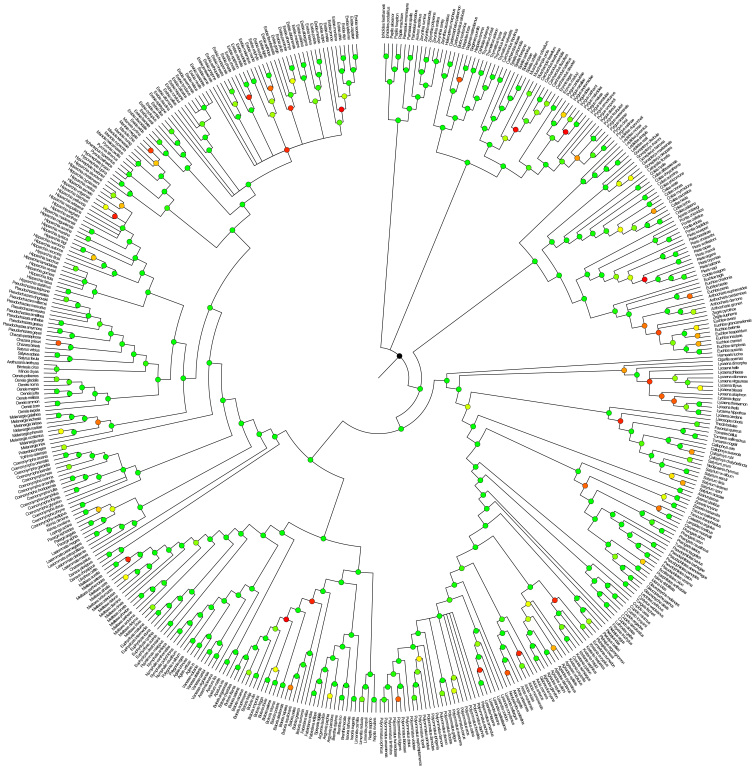
Majority rule consensus tree topology of a set of 1000 trees from the posterior distribution of time-calibrated trees of European butterflies. Circles at the nodes display clade support with a colour gradient from 50% (red) via 75% (yellow) to 100% (green).

**Figure 3.**
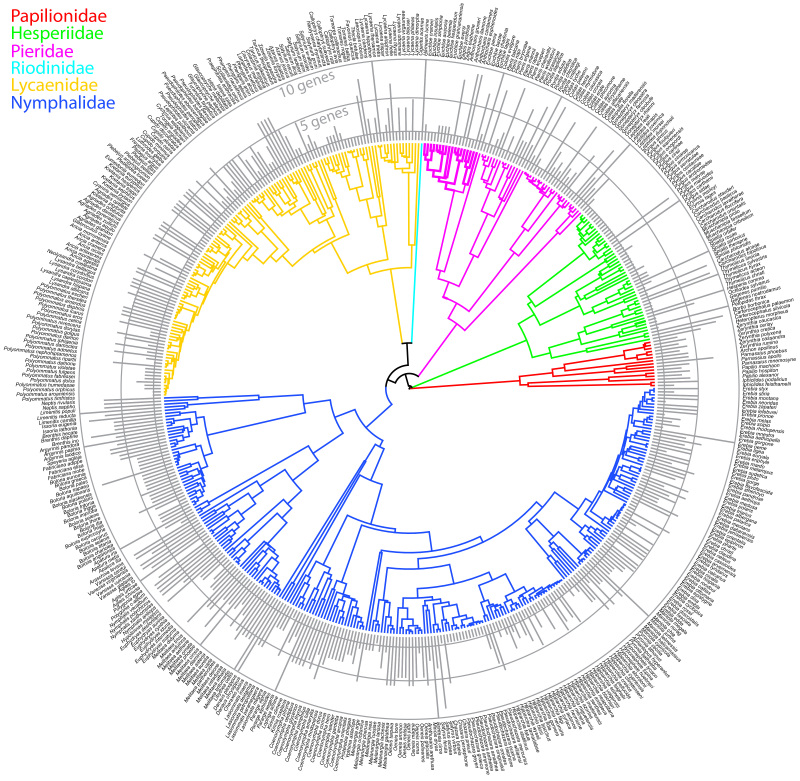
Time-calibrated tree of European butterflies. Grey bars indicate gene coverage per taxon.

We have generated a robust phylogenetic hypothesis for all European species of butterflies with estimations of divergence times (Fig. 1, Suppl. material 3: Fig. S1) as well as subtrees of major sections (Suppl. material 4: Fig. S2, Suppl. material 5: Fig. S3, Suppl. material 6: Fig. S4, Suppl. material 7: Fig. S5), a tree with posterior probabilities (Fig. 2, Suppl. material 8: Fig. S6) and gene coverage (Fig. 3). Our purpose is to provide a complete phylogenetic framework for use by the ecological and evolutionary communities. The demand for such phylogenetic information is high and various proxies have been used that are not ideal, starting already in 2005 (Päivinen et al. 2005). Although the topology of major clades in our consensus tree is largely congruent with the one by Dapporto et al. (2019), differences can be found e.g., in the monophyly of Papilioninae which appeared as a paraphylum in the trees of Dapporto et al. (2019) and Espeland et al. (2018). Our tree also confirms the monophyly of most of the European butterfly genera in the recent checklist of Wiemers et al. (2018). An exception is the genus *Carcharodus* which turned out to be a paraphylum. This result is in line with the tree in Dapporto et al. (2019) and a recent study by Zhang et al. (2020), that revises the taxonomy of Carcharodina accordingly, leading to a change of several names (Table 2). We provide a posterior distribution of topologies and node ages for researchers to be able to take phylogenetic and node age uncertainty into account in the analyses. The tree files are provided in standard Newick format as output from BEAST. Since there are easily applied methods to prune the phylogeny to the species pool of a particular study, e.g., the *ape* package (Paradis et al. 2004) in R (R Core Team 2018), our tree is readily applicable to a large variety of ecological analyses ranging from the very local and regional scales, where the species pool only represents a subset of the European species, to the European scale. Since butterflies are an important indicator taxon for biodiversity studies, this time-calibrated phylogeny will provide a solid basis to advance our understanding of large-scale biodiversity patterns and underlying mechanisms by allowing the incorporation of macro-evolutionary processes into biodiversity analyses at macroecological, landscape and local community scales and by combining trait- and phylogeny-based assessments of species assembly processes.

**Table 2.** Proposal for changes in the current taxonomic checklist by Wiemers et al. (2018) according to the recent revision of Carcharodina by Zhang et al. (2020).

**Current name (Wiemers et al. 2018)**	**Proposed name (Zhang et al. 2020)**
*Muschampia cribrellum* (Eversmann, 1841)	*Favria cribrellum* (Eversmann, 1841)
*Carcharodus lavatherae* (Esper, 1783)	Muschampia (Reverdinus) lavatherae (Esper, 1783)
*Carcharodus orientalis* Reverdin, 1913	Muschampia (Reverdinus) orientalis (Reverdin, 1913)
*Carcharodus floccifera* (Zeller, 1847)	Muschampia (Reverdinus) floccifera (Zeller, 1847)
*Carcharodus stauderi* Reverdin, 1913	Muschampia (Reverdinus) stauderi (Reverdin, 1913)
*Carcharodus baeticus* (Rambur, 1839)	Muschampia (Reverdinus) baeticus (Rambur, 1840)

## Supplementary materials

Supplementary material 1 Table listing of European butterfly species with higher taxonomy, voucher codes and accession numbers for the sequences used to build the phylogeny. Data type List of taxa with accession numbers https://binary.pensoft.net/file/418266This dataset is made available under the Open Database License (http://opendatacommons.org/licenses/odbl/1.0/). The Open Database License (ODbL) is a license agreement intended to allow users to freely share, modify, and use this Dataset while maintaining this same freedom for others, provided that the original source and author(s) are credited.Martin Wiemers, Niklas Wahlberg

Supplementary material 2 Table S1–S12 Data type PartitionFinder results Explanation note **Table S1.** PartitionFinder results for dataset 1 (Papilionidae). **Table S2.** PartitionFinder results for dataset 2 (Hesperiidae: Hesperiinae). **Table S3.** PartitionFinder results for dataset 3 (Hesperiidae: Pyrginae). **Table S4.** PartitionFinder results for dataset 4 (Pieridae). **Table S5.** Partitionfinder results for dataset 5 (Lycaenidae). **Table S6.** PartitionFinder results for dataset 6 (Nymphalidae: Danainae). **Table S7.** PartitionFinder results for dataset 7 (Nymphalidae: Apaturinae). **Table S8.** PartitionFinder results for dataset 8 (Nymphalidae: Heliconiinae + Limenitidinae). **Table S9.** PartitionFinder results for dataset 9 (Nymphalidae: Nymphalinae). **Table S10.** PartitionFinder results for dataset 10 (Nymphalidae: Satyrinae 1). **Table S11.** PartitionFinder results for dataset 11 (Nymphalidae: Satyrinae 2). **Table S12.** PartitionFinder results for dataset 12 (Nymphalidae: Satyrinae 3). https://binary.pensoft.net/file/418267This dataset is made available under the Open Database License (http://opendatacommons.org/licenses/odbl/1.0/). The Open Database License (ODbL) is a license agreement intended to allow users to freely share, modify, and use this Dataset while maintaining this same freedom for others, provided that the original source and author(s) are credited.Martin Wiemers, Nicolas Chazot, Christopher W. Wheat, Oliver Schweiger, Niklas Wahlberg

Supplementary material 3 Figure S1. Time-calibrated tree of European butterflies Data type Phylogenetic dendrogram Explanation note Unlabelled terminal branches (with monophyletic entities collapsed) represent non-European taxa which were included in the global backbone tree. Age estimates are indicated at the nodes (Ma). Node bars represent the 95% credibility intervals. https://binary.pensoft.net/file/418268This dataset is made available under the Open Database License (http://opendatacommons.org/licenses/odbl/1.0/). The Open Database License (ODbL) is a license agreement intended to allow users to freely share, modify, and use this Dataset while maintaining this same freedom for others, provided that the original source and author(s) are credited.Martin Wiemers, Nicolas Chazot, Christopher W. Wheat, Oliver Schweiger, Niklas Wahlberg

Supplementary material 4 Figure S2. Time-calibrated tree of European butterflies Section I: Papilionidae, Hesperiidae & Pieridae Data type Phylogenetic dendrogram Explanation note Age estimates are indicated at the nodes (Ma). https://binary.pensoft.net/file/418269This dataset is made available under the Open Database License (http://opendatacommons.org/licenses/odbl/1.0/). The Open Database License (ODbL) is a license agreement intended to allow users to freely share, modify, and use this Dataset while maintaining this same freedom for others, provided that the original source and author(s) are credited.Martin Wiemers, Nicolas Chazot, Christopher W. Wheat, Oliver Schweiger, Niklas Wahlberg

Supplementary material 5 Figure S3. Time-calibrated tree of European butterflies Section II. Riodinidae & Lycaenidae. Data type Phylogenetic dendrogram Explanation note Age estimates are indicated at the nodes (Ma). https://binary.pensoft.net/file/418270This dataset is made available under the Open Database License (http://opendatacommons.org/licenses/odbl/1.0/). The Open Database License (ODbL) is a license agreement intended to allow users to freely share, modify, and use this Dataset while maintaining this same freedom for others, provided that the original source and author(s) are credited.Martin Wiemers, Nicolas Chazot, Christopher W. Wheat, Oliver Schweiger, Niklas Wahlberg

Supplementary material 6 Figure S4. Time-calibrated tree of European butterflies Section III: Nymphalidae Part I: Subfamilies Limenitidinae, Heliconiinae, Apaturinae & Nymphalinae Data type Phylogenetic dendrogram Explanation note Age estimates are indicated at the nodes (Ma). https://binary.pensoft.net/file/418271This dataset is made available under the Open Database License (http://opendatacommons.org/licenses/odbl/1.0/). The Open Database License (ODbL) is a license agreement intended to allow users to freely share, modify, and use this Dataset while maintaining this same freedom for others, provided that the original source and author(s) are credited.Martin Wiemers, Nicolas Chazot, Christopher W. Wheat, Oliver Schweiger, Niklas Wahlberg

Supplementary material 7 Figure S5. Time-calibrated tree of European butterflies Section IV: Nymphalidae Part II: Subfamilies Libytheinae, Danainae & Satyrinae Data type Phylogenetic dendrogram Explanation note Age estimates are indicated at the nodes (Ma). https://binary.pensoft.net/file/418272This dataset is made available under the Open Database License (http://opendatacommons.org/licenses/odbl/1.0/). The Open Database License (ODbL) is a license agreement intended to allow users to freely share, modify, and use this Dataset while maintaining this same freedom for others, provided that the original source and author(s) are credited.Martin Wiemers, Nicolas Chazot, Christopher W. Wheat, Oliver Schweiger, Niklas Wahlberg

Supplementary material 8 Figure S6. Time-calibrated tree of European butterflies Data type Phylogenetic dendrogram Explanation note Unlabelled terminal branches (with monophyletic entities collapsed) represent non-European taxa which were included in the global backbone tree. Posterior probabilities are indicated at the nodes (Ma). https://binary.pensoft.net/file/418273This dataset is made available under the Open Database License (http://opendatacommons.org/licenses/odbl/1.0/). The Open Database License (ODbL) is a license agreement intended to allow users to freely share, modify, and use this Dataset while maintaining this same freedom for others, provided that the original source and author(s) are credited.Martin Wiemers, Nicolas Chazot, Christopher W. Wheat, Oliver Schweiger, Niklas Wahlberg

## References

[B1] Aarvik L Bengtsson BÅ Elven H Ivinskis P Jürivete U Karsholt O Mutanen M Savenkov N (2017) Nordic-Baltic Checklist of Lepidoptera. Norwegian Journal of Entomology Supplement 3: 1–237.

[B2] Aduse-Poku K Vingerhoedt E Wahlberg N (2009) Out-of-Africa again: a phylogenetic hypothesis of the genus *Charaxes* (Lepidoptera: Nymphalidae) based on five gene regions. Molecular Phylogenetics and Evolution 53: 463–478. 10.1016/j.ympev.2009.06.021 19580878

[B3] Allio R Scornavacca C Benoit N Clamens AL Sperling FAH Condamine FL (2020) Whole genome shotgun phylogenomics resolves the pattern and timing of swallowtail butterfly evolution. Systematic Biology 69: 38–60. 10.1093/sysbio/syz030 31062850

[B4] Bartonova A Benes J Konvicka M (2014) Generalist-specialist continuum and life history traits of Central European butterflies (Lepidoptera) – are we missing a part of the picture? European Journal of Entomology 111: 543–553. 10.14411/eje.2014.060

[B5] Bowler DE Haase P Kroncke I Tackenberg O Bauer HG Brendel C Brooker RW Gerisch M Henle K Hickler T Hof C Klotz S Kuhn I Matesanz S O’Hara R Russell D Schweiger O Valladares F Welk E Wiemers M Bohning-Gaese K (2015) A cross-taxon analysis of the impact of climate change on abundance trends in central Europe. Biological Conservation 187: 41–50. 10.1016/j.biocon.2015.03.034

[B6] Bowler DE Hof C Haase P Kroncke I Schweiger O Adrian R Baert L Bauer HG Blick T Brooker RW Dekoninck W Domisch S Eckmann R Hendrickx F Hickler T Klotz S Kraberg A Kuhn I Matesanz S Meschede A Neumann H O’Hara R Russell DJ Sell AF Sonnewald M Stoll S Sundermann A Tackenberg O Turkay M Valladares F van Herk K van Klink R Vermeulen R Voigtlander K Wagner R Welk E Wiemers M Wiltshire KH Bohning-Gaese K (2017) Cross-realm assessment of climate change impacts on species’ abundance trends. Nature Ecology & Evolution 1: 0067. 10.1038/s41559-016-0067 28812743

[B7] Braby MF Vila R Pierce NE (2006) Molecular phylogeny and systematics of the Pieridae (Lepidoptera: Papilionoidea): higher classification and biogeography. Zoological Journal of the Linnean Society 147: 239–275. 10.1111/j.1096-3642.2006.00264.x

[B8] Campbell DL Brower AV Pierce NE (2000) Molecular evolution of the wingless gene and its implications for the phylogenetic placement of the butterfly family Riodinidae (Lepidoptera: Papilionoidea). Molecular Biology and Evolution 17: 684–696. 10.1093/oxfordjournals.molbev.a026347 10779529

[B9] Caterino MS Reed RD Kuo MM Sperling FAH (2001) A Partitioned Likelihood Analysis of Swallowtail Phylogeny (Lepidoptera: Papilionidae). Systematic Biology 50: 106–127. 10.1080/106351501750107530 12116588

[B10] Cavender-Bares J Kozak KH Fine PVA Kembel SW (2009) The merging of community ecology and phylogenetic biology. Ecology letters 12: 693–715. 10.1111/j.1461-0248.2009.01314.x 19473217

[B11] Chazot N Wahlberg N Freitas AVL Mitter C Labandeira C Sohn J-C Sahoo RK Seraphim N de Jong R Heikkilä M (2019) Priors and Posteriors in Bayesian Timing of Divergence Analyses: the Age of Butterflies Revisited. Systematic Biology 68: 797–813. 10.1093/sysbio/syz002 30690622 PMC6893297

[B12] Condamine FL Rolland J Höhna S Sperling FAH Sanmartín I (2018) Testing the Role of the Red Queen and Court Jester as Drivers of the Macroevolution of Apollo Butterflies. Systematic Biology 67: 940–964. 10.1093/sysbio/syy009 29438538

[B13] D’Amen M Mateo RG Pottier J Thuiller W Maiorano L Pellissier L Ndiribe C Salamin N Guisan A (2018) Improving spatial predictions of taxonomic, functional and phylogenetic diversity. Journal of Ecology 106: 76–86. 10.1111/1365-2745.12801

[B14] Dapporto L Cini A Voda R Dinca V Wiemers M Menchetti M Magini G Talavera G Shreeve T Bonelli S Casacci LP Balletto E Scalercio S Vila R (2019) Integrating three comprehensive data sets shows that mitochondrial DNA variation is linked to species traits and paleogeographic events in European butterflies. Molecular Ecology Resources 19: 1623–1636. 10.1111/1755-0998.13059 31325412

[B15] Davies TJ Urban MC Rayfield B Cadotte MW Peres-Neto PR (2016) Deconstructing the relationships between phylogenetic diversity and ecology: a case study on ecosystem functioning. Ecology 97: 2212–2222. 10.1002/ecy.1507 27859062

[B16] De Palma A Kuhlmann M Bugter R Ferrier S Hoskins AJ Potts SG Roberts SPM Schweiger O Purvis A (2017) Dimensions of biodiversity loss: Spatial mismatch in land-use impacts on species, functional and phylogenetic diversity of European bees. Diversity and Distributions 23: 1435–1446. 10.1111/ddi.12638 29200933 PMC5699437

[B17] Devictor V Van Swaay C Brereton T Brotons L Chamberlain D Heliölä J Herrando S Julliard R Kuussaari M Lindström A Reif J Roy DB Schweiger O Settele J Stefanescu C Van Strien A Van Turnhout C Vermouzek S Wallis De Vries MF Wynhoff I Jiguet F (2012) Differences in the climatic debts of birds and butterflies at a continental scale. Nature Climate Change 2: 121–124. 10.1038/nclimate1347

[B18] Díaz S Purvis A Cornelissen JHC Mace GM Donoghue MJ Ewers RM Jordano P Pearse WD (2013) Functional traits, the phylogeny of function, and ecosystem service vulnerability. Ecology and Evolution 3: 2958–2975. 10.1002/ece3.601 24101986 PMC3790543

[B19] Dincă V Montagud S Talavera G Hernández-Roldán J Munguira ML García-Barros E Hebert PDN Vila R (2015) DNA barcode reference library for Iberian butterflies enables a continental-scale preview of potential cryptic diversity. Scientific Reports 5: 12395. 10.1038/srep12395 PMC4513295 26205828

[B20] Dincă V Zakharov E Hebert PD Vila R (2011) Complete DNA barcode reference library for a country’s butterfly fauna reveals high performance for temperate Europe. Proceedings of the Royal Society B 278: 347–355. 10.1098/rspb.2010.1089 20702462 PMC3013404

[B21] Drummond AJ Suchard MA Xie D Rambaut A (2012) Bayesian Phylogenetics with BEAUti and the BEAST 1.7. Molecular Biology and Evolution 29: 1969–1973. 10.1093/molbev/mss075 22367748 PMC3408070

[B22] Durka W Michalski SG (2012) Daphne: a dated phylogeny of a large European flora for phylogenetically informed ecological analyses. Ecology 93: 2297. 10.1890/12-0743.1

[B23] Economo EP Narula N Friedman NR Weiser MD Guénard B (2018) Macroecology and macroevolution of the latitudinal diversity gradient in ants. Nature Communications 9: 1778. 10.1038/s41467-018-04218-4 PMC5934361 29725049

[B24] Espeland M Breinholt J Willmott KR Warren AD Vila R Toussaint EFA Maunsell SC Aduse-Poku K Talavera G Eastwood R Jarzyna MA Guralnick R Lohman DJ Pierce NE Kawahara AY (2018) A Comprehensive and Dated Phylogenomic Analysis of Butterflies. Current Biology 28: 770–778. 10.1016/j.cub.2018.01.061 29456146

[B25] Espeland M Hall JPW DeVries PJ Lees DC Cornwall M Hsu YF Wu LW Campbell DL Talavera G Vila R Salzman S Ruehr S Lohman DJ Pierce NE (2015) Ancient Neotropical origin and recent recolonisation: Phylogeny, biogeography and diversification of the Riodinidae (Lepidoptera: Papilionoidea). Molecular Phylogenetics and Evolution 93: 296–306. 10.1016/j.ympev.2015.08.006 26265256

[B26] Essens T van Langevelde F Vos RA Van Swaay CAM Wallis De Vries MF (2017) Ecological determinants of butterfly vulnerability across the European continent. Journal of Insect Conservation 21: 439–450. 10.1007/s10841-017-9972-4

[B27] Fric ZF Maresova J Kadlec T Tropek R Pyrcz TW Wiemers M (2019) World travellers: phylogeny and biogeography of the butterfly genus *Leptotes* (Lepidoptera: Lycaenidae). Systematic Entomology 44: 652–665. 10.1111/syen.12349

[B28] Gallien L Altermatt F Wiemers M Schweiger O Zimmermann NE (2017) Invasive plants threaten the least mobile butterflies in Switzerland. Diversity and Distributions 23: 185–195. 10.1111/ddi.12513

[B29] Gerhold P Cahill JF Winter M Bartish IV Prinzing A (2015) Phylogenetic patterns are not proxies of community assembly mechanisms (they are far better). Functional Ecology 29: 600–614. 10.1111/1365-2435.12425

[B30] Hausmann A Haszprunar G Segerer AH Speidel W Behounek G Hebert PDN (2011) Now DNA-barcoded: the butterflies and larger moths of Germany. Spixiana 34: 47–58.

[B31] Heikkilä M Kaila L Mutanen M Peña C Wahlberg N (2012) Cretaceous origin and repeated tertiary diversification of the redefined butterflies. Proceedings of the Royal Society B-Biological Sciences 279: 1093–1099. 10.1098/rspb.2011.1430 PMC3267136 21920981

[B32] Huemer P Wiesmair B (2017) DNA-Barcoding der Tagfalter (Lepidoptera, Papilionoidea) Österreichs. Unbekannte genetische Vielfalt im Zentrum Europas. Wissenschaftliches Jahrbuch der Tiroler Landesmuseen 2017. StudienVerlag, Innsbruck, Wien, Bozen, 8–33.

[B33] Jetz W Thomas GH Joy JB Hartmann K Mooers AO (2012) The global diversity of birds in space and time. Nature 491: 444–448. 10.1038/nature11631 23123857

[B34] Kawahara AY Plotkin D Espeland M Meusemann K Toussaint EFA Donath A Gimnich F Frandsen PB Zwick A Dos Reis M Barber JR Peters RS Liu S Zhou X Mayer C Podsiadlowski L Storer C Yack JE Misof B Breinholt JW (2019) Phylogenomics reveals the evolutionary timing and pattern of butterflies and moths. Proceedings of the National Academy of Sciences of the Unated States of America 116(45): 22657–22663. 10.1073/pnas.1907847116 PMC6842621 31636187

[B35] Knapp S Kühn I Schweiger O Klotz S (2008) Challenging urban species diversity: contrasting phylogenetic patterns across plant functional groups in Germany. Ecology letters 11: 1054–1064. 10.1111/j.1461-0248.2008.01217.x 18616547

[B36] Kudrna O Harpke A Lux K Pennerstorfer J Schweiger O Settele J Wiemers M (2011) Distribution Atlas of Butterflies in Europe. Gesellschaft für Schmetterlingsschutz, Halle, Germany, 576 pp.

[B37] Kühn I Nobis MP Durka W (2009) Combining spatial and phylogenetic eigenvector filtering in trait analysis. Global Ecology and Biogeography 18: 745–758. 10.1111/j.1466-8238.2009.00481.x

[B38] Lanfear R Calcott B Ho SYW Guindon S (2012) PartitionFinder: Combined Selection of Partitioning Schemes and Substitution Models for Phylogenetic Analyses. Molecular Biology and Evolution 29: 1695–1701. 10.1093/molbev/mss020 22319168

[B39] Lanfear R Hua X Warren DL (2016) Estimating the Effective Sample Size of Tree Topologies from Bayesian Phylogenetic Analyses. Genome Biology and Evolution 8: 2319–2332. 10.1093/gbe/evw171 27435794 PMC5010905

[B40] Lavergne S Evans MEK Burfield IJ Jiguet F Thuiller W (2013) Are species’ responses to global change predicted by past niche evolution? Philosophical Transactions of the Royal Society B-Biological Sciences 368: 20120091. 10.1098/rstb.2012.0091 PMC3538457 23209172

[B41] Li S-P Cadotte MW Meiners SJ Hua Z-S Shu H-Y Li J-T Shu W-S (2015) The effects of phylogenetic relatedness on invasion success and impact: deconstructing Darwin’s naturalisation conundrum. Ecology letters 18: 1285–1292. 10.1111/ele.12522 26437879

[B42] Litman J Chittaro Y Birrer S Praz C Wermeille E Fluri M Stalling T Schmid S Wyler S Gonseth Y (2018) A DNA barcode reference library for Swiss butterflies and forester moths as a tool for species identification, systematics and conservation. PLoS ONE 13(12): e0208639. 10.1371/journal.pone.0208639 PMC6303096 30576327

[B43] Mazel F Pennell MW Cadotte MW Diaz S Dalla Riva GV Grenyer R Leprieur F Mooers AO Mouillot D Tucker CM Pearse WD (2018) Prioritizing phylogenetic diversity captures functional diversity unreliably. Nature Communications 9: 2888. 10.1038/s41467-018-05126-3 PMC6056549 30038259

[B44] Mazel F Renaud J Guilhaumon F Mouillot D Gravel D Thuiller W (2015) Mammalian phylogenetic diversity-area relationships at a continental scale. Ecology 96: 2814–2822. 10.1890/14-1858.1 26649401 PMC4678667

[B45] Mazel F Wüest RO Lessard J-P Renaud J Ficetola GF Lavergne S Thuiller W (2017) Global patterns of β-diversity along the phylogenetic time-scale: The role of climate and plate tectonics. Global Ecology and Biogeography 26: 1211–1221. 10.1111/geb.12632

[B46] McGeoch MA (2007) Insects and bioindication: theory and progress. In: Stewart AJA New TR Lewis OT (Eds) Conservation Biology.CABI Publishing, Oxfordshire, 144–174. 10.1079/9781845932541.0144

[B47] Monnet AC Jiguet F Meynard CN Mouillot D Mouquet N Thuiller W Devictor V (2014) Asynchrony of taxonomic, functional and phylogenetic diversity in birds. Global Ecology and Biogeography 23: 780–788. 10.1111/geb.12179 25067904 PMC4110699

[B48] Morales-Castilla I Davies TJ Pearse WD Peres-Neto P (2017) Combining phylogeny and co-occurrence to improve single species distribution models. Global Ecology and Biogeography 26: 740–752. 10.1111/geb.12580

[B49] Mouquet N Devictor V Meynard CN Munoz F Bersier LF Chave J Couteron P Dalecky A Fontaine C Gravel D Hardy OJ Jabot F Lavergne S Leibold M Mouillot D Munkemuller T Pavoine S Prinzing A Rodrigues ASL Rohr RP Thebault E Thuiller W (2012) Ecophylogenetics: advances and perspectives. Biological Reviews 87: 769–785. 10.1111/j.1469-185X.2012.00224.x 22432924

[B50] Noriega JA Hortal J Azcárate FM Berg MP Bonada N Briones MJI Del Toro I Goulson D Ibanez S Landis DA Moretti M Potts SG Slade EM Stout JC Ulyshen MD Wackers FL Woodcock BA Santos AMC (2018) Research trends in ecosystem services provided by insects. Basic and Applied Ecology 26: 8–23. 10.1016/j.baae.2017.09.006

[B51] Nylin S Wahlberg N (2008) Does plasticity drive speciation? Host-plant shifts and diversification in nymphaline butterflies (Lepidoptera: Nymphalidae) during the tertiary. Biological Journal of the Linnean Society 94: 115–130. 10.1111/j.1095-8312.2008.00964.x

[B52] Ovaskainen O Tikhonov G Norberg A Guillaume Blanchet F Duan L Dunson D Roslin T Abrego N (2017) How to make more out of community data? A conceptual framework and its implementation as models and software. Ecology Letters 20: 561–576. 10.1111/ele.12757 28317296

[B53] Päivinen J Grapputo A Kaitala V Komonen A Kotiaho JS Saarinen K Wahlberg N (2005) Negative density-distribution relationship in butterflies. BMC Biology 3: e5. 10.1186/1741-7007-3-5 PMC554103 15737240

[B54] Paradis E Claude J Strimmer K (2004) APE: Analyses of Phylogenetics and Evolution in R language. Bioinformatics 20: 289–290. 10.1093/bioinformatics/btg412 14734327

[B55] Peña C Malm T (2012) VoSeq: A Voucher and DNA Sequence Web Application. PLoS ONE 7(6): e39071. 10.1371/journal.pone.0039071 PMC3373637 22720030

[B56] Peña C Wahlberg N (2008) Prehistorical climate change increased diversification of a group of butterflies. Biology Letters 4: 274–278. 10.1098/rsbl.2008.0062 18364308 PMC2610051

[B57] Peña C Wahlberg N Weingartner E Kodandaramaiah U Nylin S Freitas AVL Brower AVZ (2006) Higher level phylogeny of Satyrinae butterflies (Lepidoptera: Nymphalidae) based on DNA sequence data. Molecular Phylogenetics and Evolution 40: 29–49. 10.1016/j.ympev.2006.02.007 16563805

[B58] Peña C Witthauer H Klečková I Fric Z Wahlberg N (2015) Adaptive radiations in butterflies: evolutionary history of the genus *Erebia* (Nymphalidae: Satyrinae). Biological Journal of the Linnean Society 116: 449–467. 10.1111/bij.12597

[B59] R Core Team (2018) R: A language and environment for statistical computing. https://www.R-project.org/ R Foundation for Statistical Computing, Vienna.

[B60] Rambaut A Drummond AJ Xie D Baele G Suchard MA (2018) Posterior Summarization in Bayesian Phylogenetics Using Tracer 1.7. Systematic Biology 67: 901–904. 10.1093/sysbio/syy032 29718447 PMC6101584

[B61] Roquet C Lavergne S Thuiller W (2014) One tree to link them all: a phylogenetic dataset for the European tetrapoda. PLoS Currents Tree of Life. 2014 Aug 8 . Edition 1. 10.1371/currents.tol.5102670fff8aa5c918e78f5592790e48 PMC4322008 25685620

[B62] Roquet C Thuiller W Lavergne S (2013) Building megaphylogenies for macroecology: taking up the challenge. Ecography 36: 13–26. 10.1111/j.1600-0587.2012.07773.x 24790290 PMC4001083

[B63] Sahoo RK Warren AD Wahlberg N Brower AVZ Lukhtanov VA Kodandaramaiah U (2016) Ten genes and two topologies: an exploration of higher relationships in skipper butterflies (Hesperiidae). PeerJ 4: e2653. 10.7717/peerj.2653 PMC5144725 27957386

[B64] Schleuning M Fründ J Schweiger O Welk E Albrecht J Albrecht M Beil M Benadi G Blüthgen N Bruelheide H Böhning-Gaese K Dehling DM Dormann CF Exeler N Farwig N Harpke A Hickler T Kratochwil A Kuhlmann M Kühn I Michez D Mudri-Stojnić S Plein M Rasmont P Schwabe A Settele J Vujić A Weiner CN Wiemers M Hof C (2016) Ecological networks are more sensitive to plant than to animal extinction under climate change. Nature Communications 7: 13965. 10.1038/ncomms13965 PMC5196430 28008919

[B65] Schweiger O Harpke A Wiemers M Settele J (2014) CLIMBER: Climatic niche characteristics of the butterflies in Europe. ZooKeys 367: 65–84. 10.3897/zookeys.367.6185 PMC3904140 24478578

[B66] Schweiger O Klotz S Durka W Kühn I (2008) A comparative test of phylogenetic diversity indices. Oecologia 157: 485–495. 10.1007/s00442-008-1082-2 18566837

[B67] Seraphim N Kaminski LA Devries PJ Penz C Callaghan C Wahlberg N Silva-Brandão KL Freitas AVL (2018) Molecular phylogeny and higher systematics of the metalmark butterflies (Lepidoptera: Riodinidae). Systematic Entomology 43: 407–425. 10.1111/syen.12282

[B68] Settele J Kudrna O Harpke A Kühn I van Swaay C Verovnik R Warren M Wiemers M Hanspach J Hickler T Kühn E van Halder I Veling K Vleigenhart A Wynhoff I Schweiger O (2008) Climatic risk atlas of European butterflies. BioRisk 1: 1–710. 10.3897/biorisk.1

[B69] Settele J Shreeve TG Konvicka M van Dyck H (2009) Ecology of Butterflies in Europe. Cambridge University Press, Cambridge, 513 pp.

[B70] Stamatakis A (2014) RAxML version 8: a tool for phylogenetic analysis and post-analysis of large phylogenies. Bioinformatics 30: 1312–1313. 10.1093/bioinformatics/btu033 24451623 PMC3998144

[B71] Stork NE (2018) How Many Species of Insects and Other Terrestrial Arthropods Are There on Earth? Annual review of entomology 63: 31–45. 10.1146/annurev-ento-020117-043348 28938083

[B72] Suchard MA Lemey P Baele G Ayres DL Drummond AJ Rambaut A (2018) Bayesian phylogenetic and phylodynamic data integration using BEAST 1.10. Virus Evolution 4: vey016. 10.1093/ve/vey016 PMC6007674 29942656

[B73] Talavera G Lukhtanov VA Pierce NE Vila R (2013) Establishing criteria for higher-level classification using molecular data: the systematics of *Polyommatus* blue butterflies (Lepidoptera, Lycaenidae). Cladistics 29: 166–192. 10.1111/j.1096-0031.2012.00421.x 34818826

[B74] Thuiller W Lavergne S Roquet C Boulangeat I Lafourcade B Araujo MB (2011) Consequences of climate change on the tree of life in Europe. Nature 470: 531–534. 10.1038/nature09705 21326204

[B75] Thuiller W Maiorano L Mazel F Guilhaumon F Ficetola GF Lavergne S Renaud J Roquet C Mouillot D (2015) Conserving the functional and phylogenetic trees of life of European tetrapods. Philosophical Transactions of the Royal Society B: Biological Sciences 370: 20140005. 10.1098/rstb.2014.0005 PMC4290419 25561666

[B76] Toussaint EFA Breinholt JW Earl C Warren AD Brower AVZ Yago M Dexter KM Espeland M Pierce NE Lohman DJ Kawahara AY (2018) Anchored phylogenomics illuminates the skipper butterfly tree of life. BMC Evolutionary Biology 18: 101. 10.1186/s12862-018-1216-z PMC6011192 29921227

[B77] Tucker CM Cadotte MW Carvalho SB Davies TJ Ferrier S Fritz SA Grenyer R Helmus MR Jin LS Mooers AO Pavoine S Purschke O Redding DW Rosauer DF Winter M Mazel F (2017) A guide to phylogenetic metrics for conservation, community ecology and macroecology. Biological Reviews 92: 698–715. ttps://10.1111/brv.12252 PMC5096690 26785932

[B78] van Swaay C Cuttelod A Collins S Maes D López Munguira ML Šašić M Settele J Verovnik R Verstrael T Warren M Wiemers M Wynhoff I (2010) European Red List of Butterflies. Publications Office of the European Union, Luxembourg, 46 pp.

[B79] van Swaay C Warren M Lois G (2006) Biotope use and trends of European butterflies. Journal of Insect Conservation 10: 305–306. 10.1007/s10841-006-8361-1

[B80] Verovnik R Wiemers M (2016) Species delimitation in the Grayling genus *Pseudochazara* (Lepidoptera, Nymphalidae, Satyrinae) supported by DNA barcodes. ZooKeys 600: 131–154. 10.3897/zookeys.600.7798 PMC4926685 27408604

[B81] Vila R Lukhtanov VA Talavera G Gil TF Pierce NE (2010) How common are dot-like distributions? Taxonomical oversplitting in western European *Agrodiaetus* (Lepidoptera: Lycaenidae) revealed by chromosomal and molecular markers. Biological Journal of the Linnean Society 101: 130–154. 10.1111/j.1095-8312.2010.01481.x

[B82] Vishnevskaya MS Saifitdinova AF Lukhtanov VA (2016) Karyosystematics and molecular taxonomy of the anomalous blue butterflies (Lepidoptera, Lycaenidae) from the Balkan Peninsula. Comparative Cytogenetics 10: 1–85. 10.3897/CompCytogen.v10i5.10944 PMC5220643 28105291

[B83] Wahlberg N Braby MF Brower AVZ de Jong R Lee MM Nylin S Pierce NE Sperling FAH Vila R Warren AD Zakharov E (2005a) Synergistic effects of combining morphological and molecular data in resolving the phylogeny of butterflies and skippers. Proceedings of the Royal Society B-Biological Sciences 272: 1577–1586. 10.1098/rspb.2005.3124 PMC1560179 16048773

[B84] Wahlberg N Brower AVZ Nylin S (2005b) Phylogenetic relationships and historical biogeography of tribes and genera in the subfamily Nymphalinae (Lepidoptera: Nymphalidae). Biological Journal of the Linnean Society 86: 227–251. 10.1111/j.1095-8312.2005.00531.x

[B85] Wahlberg N Leneveu J Kodandaramaiah U Peña C Nylin S Freitas AVL Brower AVZ (2009) Nymphalid butterflies diversify following near demise at the Cretaceous/Tertiary boundary. Proceedings of the Royal Society B-Biological Sciences 276: 4295–4302. 10.1098/rspb.2009.1303 PMC2817107 19793750

[B86] Wahlberg N Rota J Braby MF Pierce NE Wheat CW (2014) Revised systematics and higher classification of pierid butterflies (Lepidoptera: Pieridae) based on molecular data. Zoologica Scripta 43: 641–650. 10.1111/zsc.12075

[B87] Wahlberg N Weingartner E Nylin S (2003) Towards a better understanding of the higher systematics of Nymphalidae (Lepidoptera: Papilionoidea). Molecular Phylogenetics and Evolution 28: 473–484. 10.1016/S1055-7903(03)00052-6 12927132

[B88] Wahlberg N Wheat CW (2008) Genomic outposts serve the phylogenomic pioneers: Designing novel nuclear markers for genomic DNA extractions of lepidoptera. Systematic Biology 57: 231–242. 10.1080/10635150802033006 18398768

[B89] Warren AD Ogawa JR Brower AVZ (2008) Phylogenetic relationships of subfamilies and circumscription of tribes in the family Hesperiidae (Lepidoptera : Hesperioidea). Cladistics 24: 642–676. 10.1111/j.1096-0031.2008.00218.x

[B90] Webb CO Ackerly DD McPeek MA Donoghue MJ (2002) Phylogenies and community ecology. Annual Review of Ecology and Systematics 33: 475–505. 10.1146/annurev.ecolsys.33.010802.150448

[B91] Wiemers M Balletto E Dinca V Faltynek Fric Z Gerardo L Lukhtanov V Munguira ML Chris AMvS Vila R Vliegenthart A Wahlberg N Verovnik R (2018) An updated checklist of the European Butterflies (Lepidoptera, Papilionoidea). ZooKeys 811: 9–45. 10.3897/zookeys.811.28712 PMC6323101 30627036

[B92] Wiemers M Fiedler K (2007) Does the DNA barcoding gap exist? - a case study in blue butterflies (Lepidoptera: Lycaenidae). Frontiers in Zoology 4: 8. 10.1186/1742-9994-4-8 PMC1838910 17343734

[B93] Wiemers M Stradomsky BV Vodolazhsky DI (2010) A molecular phylogeny of *Polyommatus* s. str. and *Plebicula* based on mitochondrial COI and nuclear ITS2 sequences (Lepidoptera: Lycaenidae). European Journal of Entomology 107: 325–336. 10.14411/eje.2010.041

[B94] Wiens JJ Graham CH (2005) Niche Conservatism: Integrating Evolution, Ecology, and Conservation Biology. Annual Review of Ecology, Evolution, and Systematics 36: 519–539. 10.1146/annurev.ecolsys.36.102803.095431

[B95] Winter M Devictor V Schweiger O (2013) Phylogenetic diversity and nature conservation: where are we? Trends in Ecology & Evolution 28: 199–204. 10.1016/j.tree.2012.10.015 23218499

[B96] Winter M Schweiger O Klotz S Nentwig W Andriopoulos P Arianoutsou M Basnou C Delipetrou P Didziulis V Hejda M Hulme PE Lambdon PW Pergl J Pyšek P Roy DB Kühn I (2009) Plant extinctions and introductions lead to phylogenetic and taxonomic homogenization of the European flora. Proceedings of the National Academy of Sciences of the United States of America 106: 21721–21725. 10.1073/pnas.0907088106 20007367 PMC2792159

[B97] Zhang J Brockmann E Cong Q Shen J Grishin NV (2020) A genomic perspective on the taxonomy of the subtribe Carcharodina (Lepidoptera: Hesperiidae: Carcharodini). Zootaxa 4748: 182–194. 10.11646/zootaxa.4748.1.10 PMC8018707 32230093

