# Supplementary material for: A complete time-calibrated multi-gene phylogeny of the European butterflies
Source: Zookeys. 2020 Jun 4;938:97–124. doi: 10.3897/zookeys.938.50878 (PMC7289901; doi:10.3897/zookeys.938.50878)
Supplement: Supplementary material 2 — Table S1–S12 [file zookeys-938-097-s002.docx]

# Table S 1. PartitionFinder results for dataset 1 (Papilionidae)

| Subset # | Substitution model | Gene fragments and codon positions |
| --- | --- | --- |
| Subset 1 | TRN+I+G | ArgK_pos1, RpS2_pos1, EF-1α_pos1, RpS5_pos1 |
| Subset 2 | TRN+I+G | COI-begin_pos2, RpS5_pos2, ArgK_pos2, GAPDH_pos2, CAD_pos2, MDH_pos2, IDH_pos2, COI-end_pos2 |
| Subset 3 | HKY | ArgK_pos3 |
| Subset 4 | HKY+I+G | CAD_pos3 |
| Subset 5 | GTR+G | wingless_pos1, IDH_pos1, GAPDH_pos1, MDH_pos1, CAD_pos1 |
| Subset 6 | TRN+G | COI-end_pos3, COI-begin_pos3 |
| Subset 7 | GTR+G | DDC_pos2, COI-end_pos1, COI-begin_pos1 |
| Subset 8 | GTR+I+G | DDC_pos3, wingless_pos3, RpS2_pos3 |
| Subset 9 | K80+G | DDC_pos1, wingless_pos2 |
| Subset 10 | GTR+G | EF-1α_pos3 |
| Subset 11 | TRNEF+I | RpS2_pos2, EF-1α_pos2 |
| Subset 12 | GTR+G | RpS5_pos3, GAPDH_pos3, IDH_pos3, MDH_pos3 |

# Table S 2. PartitionFinder results for dataset 2 (Hesperiidae: Hesperiinae)

| Subset # | Substitution model | Gene fragments and codon positions |
| --- | --- | --- |
| Subset 1 | GTR+I+G | COI-end_pos1, COI-begin_pos1, ArgK_pos1 |
| Subset 2 | GTR+I+G | ArgK_pos2, IDH_pos2, EF-1α_pos2 |
| Subset 3 | GTR+G | ArgK_pos3 |
| Subset 4 | TRN+I+G | CAD_pos3, IDH_pos3 |
| Subset 5 | GTR+I+G | IDH_pos1, CAD_pos1 |
| Subset 6 | GTR+I+G | CAD_pos2, MDH_pos2 |
| Subset 7 | TRN+I+G | COI-begin_pos3, COI-end_pos3 |
| Subset 8 | HKY+I+G | RpS5_pos2, COI-begin_pos2, GAPDH_pos2, COI-end_pos2 |
| Subset 9 | GTR+I+G | EF-1α_pos3 |
| Subset 10 | GTR+I+G | EF-1α_pos1, MDH_pos1 |
| Subset 11 | GTR+I+G | RpS2_pos3, GAPDH_pos3, RpS5_pos3 |
| Subset 12 | GTR+I+G | GAPDH_pos1, wingless_pos2 |
| Subset 13 | TRN+I+G | MDH_pos3 |
| Subset 14 | JC+I | RpS2_pos2 |
| Subset 15 | GTR+I+G | RpS2_pos1, RpS5_pos1 |
| Subset 16 | GTR+I+G | wingless_pos3 |
| Subset 17 | SYM+I+G | wingless_pos1 |

# Table S 3. PartitionFinder results for dataset 3 (Hesperiidae: Pyrginae)

| Subset # | Substitution model | Gene fragments and codon positions |
| --- | --- | --- |
| Subset 1 | TRN+I+G | EF-1α_pos1, IDH_pos1, RpS2_pos1, RpS5_pos1, ArgK_pos1 |
| Subset 2 | TRN+I+G | EF-1α_pos2, GAPDH_pos2, MDH_pos2, COI-begin_pos2, COI-end_pos2, IDH_pos2, CAD_pos2, RpS5_pos2, ArgK_pos2 |
| Subset 3 | GTR+G | ArgK_pos3 |
| Subset 4 | HKY+I+G | CAD_pos3 |
| Subset 5 | GTR+I+G | CAD_pos1, MDH_pos1, GAPDH_pos1 |
| Subset 6 | HKY+G | COI-begin_pos3 |
| Subset 7 | GTR+I+G | COI-end_pos1, COI-begin_pos1 |
| Subset 8 | TRN+I+G | COI-end_pos3 |
| Subset 9 | GTR+G | EF-1α_pos3 |
| Subset 10 | GTR+I+G | RpS2_pos3, GAPDH_pos3, RpS5_pos3 |
| Subset 11 | GTR+I+G | MDH_pos3, IDH_pos3 |
| Subset 12 | JC+G | wingless_pos2, RpS2_pos2 |
| Subset 13 | HKY+G | wingless_pos3 |
| Subset 14 | SYM+G | wingless_pos1 |

# Table S 4. PartitionFinder results for dataset 4 (Pieridae)

| Subset # | Substitution model | Gene fragments and codon positions |
| --- | --- | --- |
| Subset 1 | GTR+I+G | ArgK_pos1, EF-1α_pos1, GAPDH_pos1 |
| Subset 2 | TRN+I+G | ArgK_pos2, DDC_pos2, CAD_pos2, IDH_pos2 |
| Subset 3 | KHY | ArgK_pos3 |
| Subset 4 | HKY+I+G | CAD_pos3 |
| Subset 5 | GTR+I+G | CAD_pos1, RpS2_pos1, IDH_pos1, RpS5_pos1, MDH_pos1 |
| Subset 6 | HKY+G | COI-begin_pos3 |
| Subset 7 | GTR+I+G | COI-begin_pos1, COI-end_pos1 |
| Subset 8 | GTR+I+G | MDH_pos2, COI-begin_pos2, RpS5_pos2, COI-end_pos2, EF-1α_pos2, GAPDH_pos2 |
| Subset 9 | TRN+I+G | COI-end_pos3 |
| Subset 10 | K80+G | DDC_pos3, RpS2_pos3 |
| Subset 11 | SYM+G | DDC_pos1, wingless_pos1 |
| Subset 12 | GTR+I+G | EF-1α_pos3 |
| Subset 13 | GTR+I+G | RpS5_pos3, GAPDH_pos3 |
| Subset 14 | GTR+I+G | IDH_pos3, MDH_pos3 |
| Subset 15 | JC+I | RpS2_pos2 |
| Subset 16 | GTR+I+G | wingless_pos3 |
| Subset 17 | K80+G | wingless_pos2 |

# Table S 5. Partitionfinder results for dataset 5 (Lycaenidae)

| Subset # | Substitution model | Gene fragments and codon positions |
| --- | --- | --- |
| Subset 1 | TRN+I+G | ArgK_pos1, H3_pos1, EF-1α_pos1, RpS5_pos1, GAPDH_pos1 |
| Subset 2 | GTR+I+G | RpS5_pos2, GAPDH_pos2, ArgK_pos2, DDC_pos2, MDH_pos2, COI-begin_pos2, COI-end_pos2, EF-1α_pos2, IDH_pos2, CAD_pos2 |
| Subset 3 | GTR+G | H3_pos3, ArgK_pos3, wingless_pos3 |
| Subset 4 | HKY+I+G | CAD_pos3 |
| Subset 5 | GTR+G | wingless_pos2, RpS2_pos1, MDH_pos1, IDH_pos1, CAD_pos1 |
| Subset 6 | GTR+G | COI-begin_pos3, COI-end_pos3 |
| Subset 7 | GTR+I+G | COI-begin_pos1, COI-end_pos1 |
| Subset 8 | TRNEF | DDC_pos3, RpS2_pos3 |
| Subset 9 | SYM+G | wingless_pos1, DDC_pos1 |
| Subset 10 | GTR+I+G | EF-1α_pos3 |
| Subset 11 | GTR+G | MDH_pos3, RpS5_pos3, GAPDH_pos3, IDH_pos3 |
| Subset 12 | JC+I | RpS2_pos2, H3_pos2 |

# Table S 6. PartitionFinder results for dataset 6 (Nymphalidae: Danainae)

| Subset # | Substitution model | Gene fragments and codon positions |
| --- | --- | --- |
| Subset 1 | HKY+I | MDH_pos3, CAD_pos3 |
| Subset 2 | GTR+G | RpS5_pos1, RpS2_pos1, GAPDH_pos1, EF-1α_pos1, wingless_pos1, MDH_pos1, CAD_pos1, IDH_pos1 |
| Subset 3 | HKY+I | COI-end_pos2, COI-begin_pos2, RpS5_pos2, GAPDH_pos2, MDH_pos2, EF-1α_pos2, CAD_pos2, IDH_pos2 |
| Subset 4 | HKY+G | COI-end_pos3, COI-begin_pos3 |
| Subset 5 | TRN+G | COI-end_pos1, COI-begin_pos1 |
| Subset 6 | HKY+G | EF-1α_pos3, RpS5_pos3 |
| Subset 7 | GTR+G | GAPDH_pos3, RpS2_pos3, IDH_pos3, wingless_pos3 |
| Subset 8 | JC | RpS2_pos2, wingless_pos2 |

# Table S 7. PartitionFinder results for dataset 7 (Nymphalidae: Apaturinae)

| Subset # | Substitution model | Gene fragments and codon positions |
| --- | --- | --- |
| Subset 1 | TRN+G | wingless_pos1, CAD_pos1, IDH_pos1, ArgK_pos1, RpS5_pos1, RpS2_pos1, EF-1α_pos1, MDH_pos1, GAPDH_pos1 |
| Subset 2 | GTR+I | COI-begin_pos2, EF-1α_pos2, ArgK_pos2, MDH_pos2, CAD_pos2, COI-end_pos2, IDH_pos2, RpS5_pos2, GAPDH_pos2 |
| Subset 3 | GTR+G | EF-1α_pos3, RpS2_pos3, RpS5_pos3, GAPDH_pos3, ArgK_pos3, wingless_pos3 |
| Subset 4 | HKY+I | IDH_pos3, MDH_pos3, CAD_pos3 |
| Subset 5 | HKY+G | COI-end_pos3, COI-begin_pos3 |
| Subset 6 | TRN+G | COI-end_pos1, COI-begin_pos1 |
| Subset 7 | JC | RpS2_pos2, wingless_pos2 |

# Table S 8. PartitionFinder results for dataset 8 (Nymphalidae: Heliconiinae + Limenitidinae)

| Subset # | Substitution model | Gene fragments and codon positions |
| --- | --- | --- |
| Subset 1 | GTR+I+G | wingless_pos2, RpS2_pos1, ArgK_pos1 |
| Subset 2 | GTR+I+G | DDC_pos2, IDH_pos2, GAPDH_pos2, RpS5_pos2, EF-1α_pos2, ArgK_pos2 |
| Subset 3 | GTR+G | ArgK_pos3 |
| Subset 4 | HKY+I+G | MDH_pos3, CAD_pos3 |
| Subset 5 | GTR+I+G | DDC_pos1, wingless_pos1, CAD_pos1, RpS5_pos1, MDH_pos1, IDH_pos1 |
| Subset 6 | GTR+I+G | CAD_pos2, MDH_pos2, COI-begin_pos2, COI-end_pos2 |
| Subset 7 | GTR+I+G | COI-end_pos3, COI-begin_pos3 |
| Subset 8 | GTR+I+G | COI-end_pos1, COI-begin_pos1 |
| Subset 9 | GTR+I+G | DDC_pos3, wingless_pos3, RpS5_pos3, RpS2_pos3 |
| Subset 10 | GTR+I+G | EF-1α_pos3 |
| Subset 11 | TRN+I+G | EF-1α_pos1, GAPDH_pos1 |
| Subset 12 | GTR+I+G | GAPDH_pos3 |
| Subset 13 | GTR+I+G | IDH_pos3 |
| Subset 14 | JC+I | RpS2_pos2 |

# Table S 9. PartitionFinder results for dataset 9 (Nymphalidae: Nymphalinae)

| Subset # | Substitution model | Gene fragments and codon positions |
| --- | --- | --- |
| Subset 1 | TRN+I+G | wingless_pos1, ArgK_pos1, MDH_pos1, CAD_pos1, IDH_pos1, GAPDH_pos1 |
| Subset 2 | GTR+I+G | DDC_pos2, IDH_pos2, ArgK_pos2, EF-1α_pos2, RpS5_pos2, MDH_pos2, CAD_pos2 |
| Subset 3 | GTR+G | EF-1α_pos3, ArgK_pos3 |
| Subset 4 | HKY+I+G | MDH_pos3, CAD_pos3 |
| Subset 5 | GTR+G | COI-end_pos3, COI-begin_pos3 |
| Subset 6 | GTR+G | COI-end_pos1, COI-begin_pos1 |
| Subset 7 | GTR+I+G | COI-begin_pos2, COI-end_pos2 |
| Subset 8 | TRNEF | wingless_pos3, DDC_pos3 |
| Subset 9 | SYM+G | DDC_pos1, wingless_pos2 |
| Subset 10 | GTR+I+G | RpS5_pos1, RpS2_pos1, EF-1α_pos1 |
| Subset 11 | GTR+G | IDH_pos3, RpS2_pos3, GAPDH_pos3, RpS5_pos3 |
| Subset 12 | JC+I | RpS2_pos2, GAPDH_pos2 |

# Table S 10. PartitionFinder results for dataset 10 (Nymphalidae: Satyrinae 1)

| Subset # | Substitution model | Gene fragments and codon positions |
| --- | --- | --- |
| Subset 1 | HKY+G | COI-begin_pos3, COI-end_pos3 |
| Subset 2 | TRN+G | COI-begin_pos1, COI-end_pos1 |
| Subset 3 | HKY+I | EF-1α_pos2, RpS5_pos2, GAPDH_pos2, COI-end_pos2, COI-begin_pos2 |
| Subset 4 | GTR+G | GAPDH_pos3, EF-1α_pos3, RpS5_pos3 |
| Subset 5 | TRN+G | wingless_pos2, wingless_pos1, GAPDH_pos1, EF-1α_pos1, RpS5_pos1 |
| Subset 6 | GTR | wingless_pos3 |

# Table S 11. PartitionFinder results for dataset 11 (Nymphalidae: Satyrinae 2)

| Subset # | Substitution model | Gene fragments and codon positions |
| --- | --- | --- |
| Subset 1 | GTR+I+G | ArgK_pos1, wingless_pos1, RpS2_pos1, IDH_pos1, CAD_pos1, EF-1α_pos1, GAPDH_pos1, RpS5_pos1, MDH_pos1 |
| Subset 2 | HKY+I+G | MDH_pos2, ArgK_pos2, CAD_pos2, IDH_pos2, RpS5_pos2, EF-1α_pos2, GAPDH_pos2 |
| Subset 3 | GTR+G | ArgK_pos3, wingless_pos3 |
| Subset 4 | HKY+G | IDH_pos3, MDH_pos3, CAD_pos3 |
| Subset 5 | GTR+G | COI-begin_pos3, COI-end_pos3 |
| Subset 6 | GTR+G | COI-begin_pos1, COI-end_pos1 |
| Subset 7 | TRN+I+G | COI-begin_pos2, COI-end_pos2 |
| Subset 8 | GTR+I+G | EF-1α_pos3, RpS5_pos3 |
| Subset 9 | HKY+G | GAPDH_pos3 |
| Subset 10 | TRNEF+G | RpS2_pos2, wingless_pos2 |
| Subset 11 | K80+G | RpS2_pos3 |

# Table S 12. PartitionFinder results for dataset 12 (Nymphalidae: Satyrinae 3)

| Subset # | Substitution model | Gene fragments and codon positions |
| --- | --- | --- |
| Subset 1 | HKY | CAD_pos3 |
| Subset 2 | TRN+G | RpS2_pos2, COI-begin_pos1, COI-end_pos1, CAD_pos1, IDH_pos1 |
| Subset 3 | HKY | RpS5_pos2, MDH_pos2, GAPDH_pos2, EF-1α_pos2, IDH_pos2, CAD_pos2 |
| Subset 4 | GTR+G | COI-end_pos3, COI-begin_pos3 |
| Subset 5 | HKY+I | COI-end_pos2, COI-begin_pos2 |
| Subset 6 | HKY+G | wingless_pos3, RpS2_pos3, EF-1α_pos3 |
| Subset 7 | TRN+G | wingless_pos1, RpS2_pos1, MDH_pos1, GAPDH_pos1, RpS5_pos1, EF-1α_pos1 |
| Subset 8 | HKY+G | RpS5_pos3, MDH_pos3, GAPDH_pos3, IDH_pos3 |
| Subset 9 | JC | wingless_pos2 |
