# Supplementary figures and images for: A complete time-calibrated multi-gene phylogeny of the European butterflies
Source: Zookeys. 2020 Jun 4;938:97–124. doi: 10.3897/zookeys.938.50878 (PMC7289901; doi:10.3897/zookeys.938.50878)

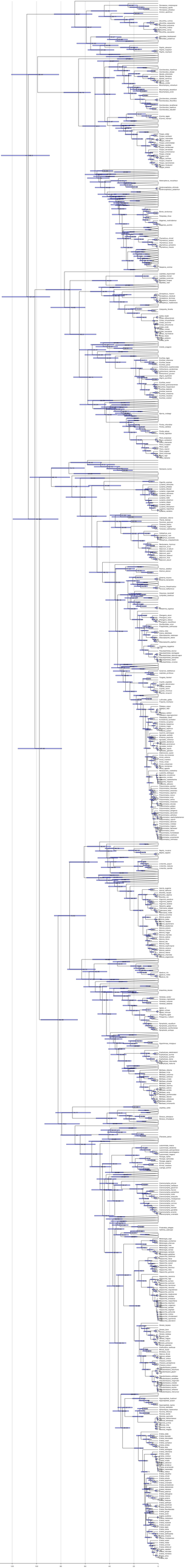

Supplement: Supplementary material 3 — Figure S1. Time-calibrated tree of European butterflies [file zookeys-938-097-s003.pdf]

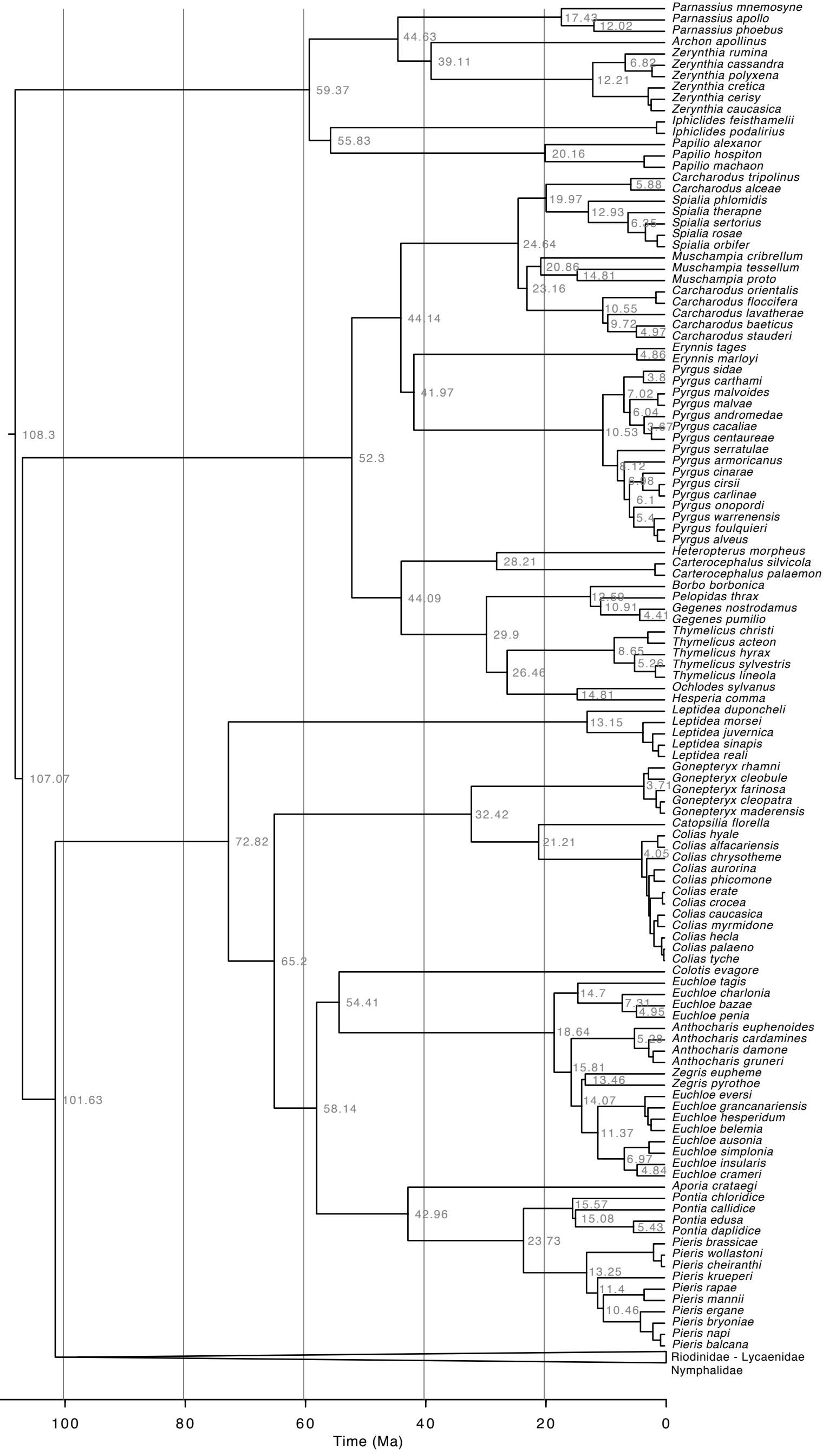

Supplement: Supplementary material 4 — Figure S2. Time-calibrated tree of European butterflies Section I: Papilionidae, Hesperiidae & Pieridae [file zookeys-938-097-s004.pdf]

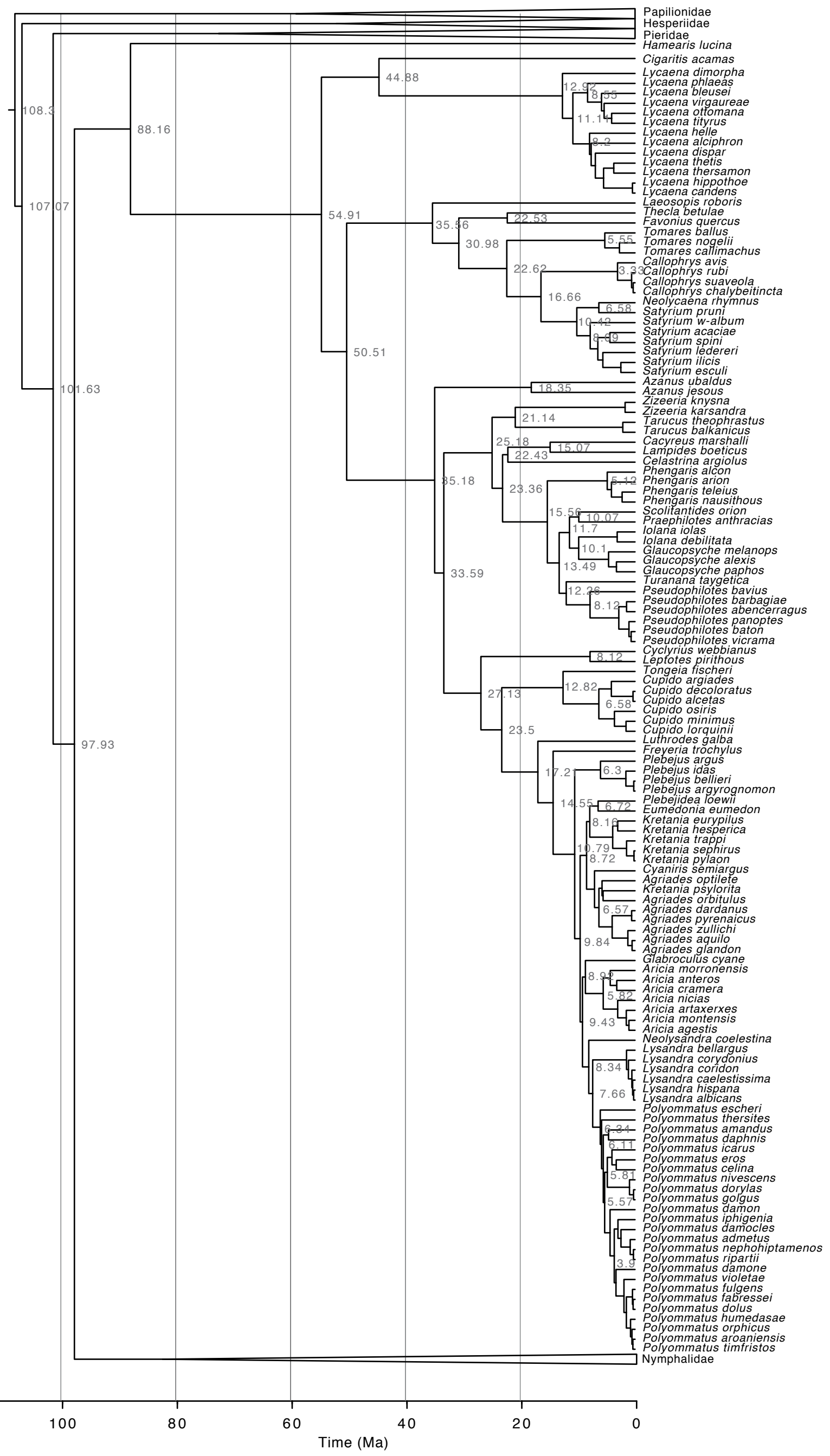

Supplement: Supplementary material 5 — Figure S3. Time-calibrated tree of European butterflies Section II. Riodinidae & Lycaenidae. [file zookeys-938-097-s005.pdf]

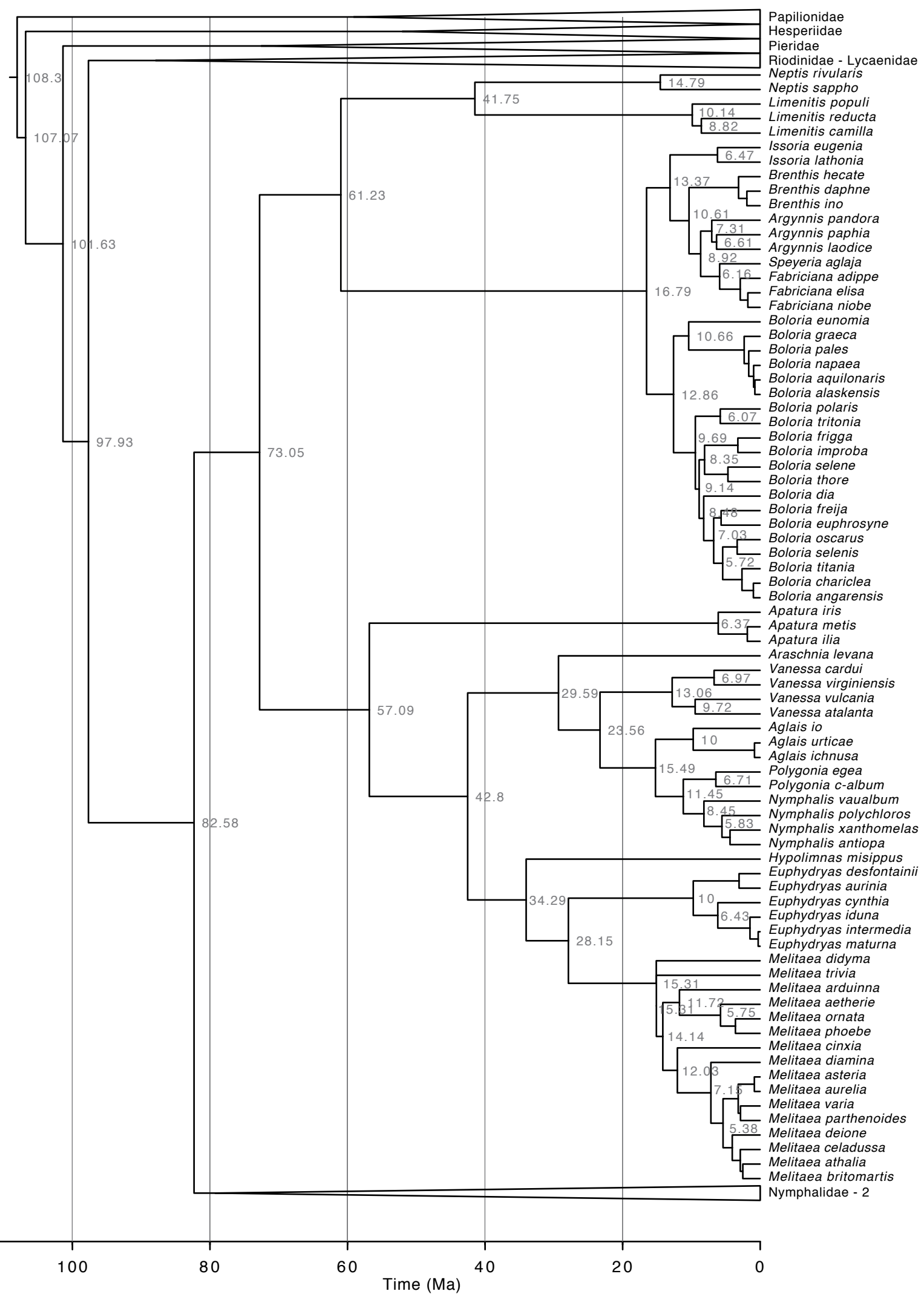

Supplement: Supplementary material 6 — Figure S4. Time-calibrated tree of European butterflies Section III: Nymphalidae Part I: Subfamilies Limenitidinae, Heliconiinae, Apaturinae & Nymphalinae [file zookeys-938-097-s006.pdf]

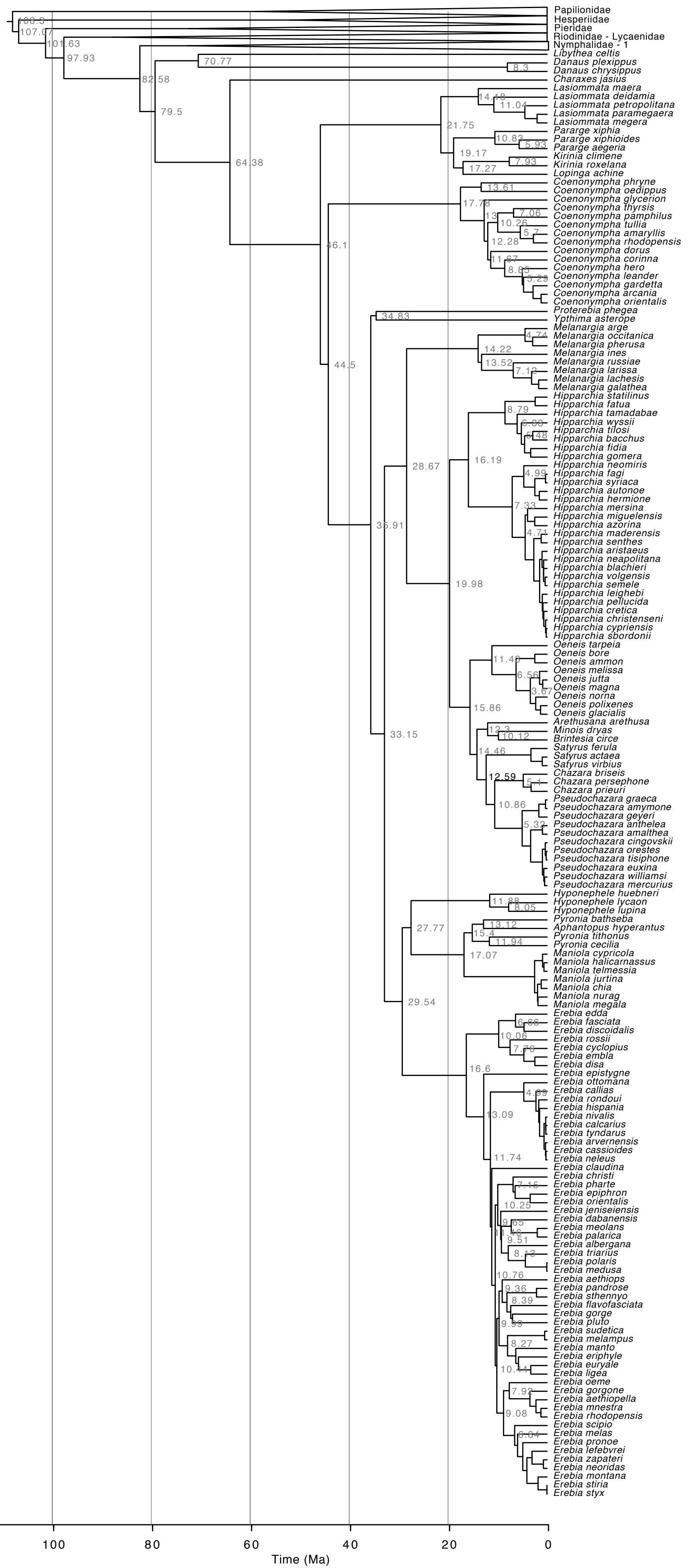

Supplement: Supplementary material 7 — Figure S5. Time-calibrated tree of European butterflies Section IV: Nymphalidae Part II: Subfamilies Libytheinae, Danainae & Satyrinae [file zookeys-938-097-s007.pdf]

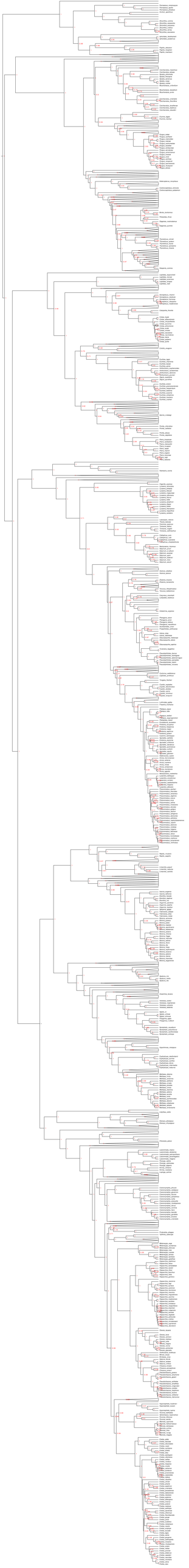

Supplement: Supplementary material 8 — Figure S6. Time-calibrated tree of European butterflies [file zookeys-938-097-s008.pdf]
